# Antibacterial, antibiofilm, and antiproliferative properties of *Aspergillus frequens*-derived pigment

**DOI:** 10.1186/s12934-025-02888-6

**Published:** 2025-12-27

**Authors:** Asmaa S. Yassein, Osama A. M. Al-Bedak, Rokaia B. Elamary

**Affiliations:** 1 Department of Botany and Microbiology, Faculty of Science, Qena University, Qena, 83523 Egypt; 2 Assiut University Mycological Centre, Assiut, 71511 Egypt; 3 Science & Innovation Center of Excellent, Egyptian Russian University, Badr city, Cairo, 11829 Egypt; 4 Faculty of Science, Botany and Microbiology Department, Luxor University, Luxor, Egypt

**Keywords:** Pigmented secondary metabolites, FTIR, GC‒MS, Biofilm, Zeta potential, Bone and lung cancer

## Abstract

**Background:**

Filamentous fungi produce a broad spectrum of colored secondary metabolites that are largely used in various industries, including food, cosmetics, fabrics, and medications. This study explores, for the first time, the potential of *Aspergillus frequens* to produce pigmented secondary metabolites and their application in various biotechnological treatments.

**Results:**

*Aspergillus frequens* (Asmaa 2024) produced the highest concentration of pigmented secondary metabolites among the 20 tested fungal rhizospheric fungi, reaching 21.36 ± 1.8 AU/mL in potato dextrose broth (PDB) medium. Scanning electron microscopy (SEM) revealed that the extracted pigment has an irregular shape and particle size, ranging from 40 to 184 nm. The elemental composition revealed the presence of high ratios of carbon and oxygen using energy-dispersive X-ray (EDX). Many functional groups and chromophore compounds have been detected in the extracted pigment using Fourier-transform infrared spectroscopy (FT-IR) and gas chromatography–mass spectrometry (GC-MS). Thirteen pathogenic species of bacteria were significantly inhibited in their development by the colored metabolites, whose minimum bactericidal concentrations (MBCs) varied from 4.5 to 16.7 mg/mL. The most notable percentages in suppression biofilm development, suggesting a major influence, were 66.8% for *Klebsiella pneumoniae* and 64.8% for *Bacillus subtilis* using the microtiter plate technique. Following assessment of zeta potential, particle size, and polydispersity index (PDI) of the target bacteria, the effective antibacterial efficacy of the pigmented secondary metabolites was confirmed. The viability of the osteosarcoma (HOS) and lung cancer (A549) cell lines was significantly diminished by the *A. frequens*’ secondary metabolites, with IC50 values of 43.3 and 77.1 µg/mL, respectively. In contrast, the skin cancer cell line (A431) showed no signs of impact, using the MTT assay.

**Conclusion:**

Based on the obtained findings, *A. frequens* pigmented secondary metabolites have promising potential in the biological control of pathogenic and biofilm-forming bacteria, as well as in the treatment of bone and lung cancer. While numerous studies have investigated pigment production in *Aspergillus* species, this research represents the first investigation into pigment synthesis by *A. frequens*.

## Introduction

Pigments are compounds responsible for yielding color to both organic and inorganic materials due to their ability to selectively absorb restricted wavelengths of light. They are usually classified into two main types: natural and synthetic pigments [1]. Interest in creating natural pigments has grown worldwide as a result of the dangers of the majority of synthetic colorants and the urgent need for non-toxic, non-carcinogenic, non-poisoning, and eco-friendly alternatives for use in food, cosmetics, medicines, environmental bioremediation, and textiles to overcome the health hazards linked to synthetic coloring agents, including allergies, mental health issues, and different forms of cancer [1–4]. Colorants are used by a variety of industries to alter or enhance the color of various substrates [5]. Numerous biological sources, such as microorganisms, animals, insects, and plants, can provide colored substances, and till now, microbes are more satisfactory than other living individuals [6, 7]. Because of their sustainability and economic feasibility as natural colorant sources, filamentous fungi are a major area of industrial research and are capable of producing a variety of widely used pigments [8].

Secondary metabolites called fungal pigments are occasionally created as a result of nutritional value shortages. Secondary metabolites are produced by mycelium in response to a decline in the nutritional supply of critical nutrients or an unfavorable environmental situation [9]. *Aspergillus*, *Fusarium*, *Penicillium*, and *Trichoderma* are among the fungi that, as they grow, create a variety of colors as intermediate metabolites [10]. Fungal pigments fall into two categories: polyketides and carotenoids. Tetraketides and octaketides, which have eight C_2_ units and form polyketide chains, are the building blocks of fungal polyketides. A distinct class of fungal polyketides known as azaphilones is distinguished by its pyrano-quinone structures with high electron-accepting capacity, which influences the oxygen sensitivity of the primary ring and includes a chiral center. This results in the formation of γ-pyridones with chromophoric properties, where the color is determined by their chemical structure [11, 12]. Three requirements must be met for the use of filamentous fungal biopigments in associated products: the pigment molecules’ stability and purity, the lack of potentially harmful elements, and a profitable manufacturing yield [13]. Biopigments have been described to have antibacterial, anti-inflammatory, antioxidant, and anticancer effects [14].

Bacteria can develop biofilms, which enclose them in extracellular polymeric substances (EPSs) and increase their resistance to antimicrobial therapy. This is a significant health concern [15, 16]. The development of alternative non-antibiotic techniques, such as the use of natural plant extracts and enzymes, is crucial for effectively targeting infectious bacteria while preserving the health of humans and animals [16–18]. For many years, antimicrobials made from fungi have been used [19]. Metabolites with antimicrobial properties, including phenols, alkaloids, polyketides, steroids, terpenoids, flavonoids, polysaccharides, aliphatic compounds, and quinones, are produced by filamentous fungi [20].

Thus, this study aims to determine the potential of 20 fungal isolates from rhizospheric soil to generate colored secondary metabolites. ITS sequencing was utilized to confirm the morphological classification of *Aspergillus frequens* strain Asmaa 2024, which was shown to be the most active strain for pigment production. Despite the profusion of studies verifying pigment production in various *Aspergillus* species, there is nevertheless no investigation specifically targeting *A. frequens* pigments. The pigment characterization was performed using UV‒Visible Spectrophotometry, FTIR, GC-MS spectrophotometry, elemental analysis, SEM, and EDX. The antibacterial, antibiofilm, as well as anticancer activities against osteosarcoma (HOS), lung carcinoma (A549), and skin cancer cell lines (A431) were also confirmed. Despite the profusion of studies verifying pigment production in various *Aspergillus* species, there is nevertheless no investigation specifically targeting *A. frequens* pigments and their biological activities. Future studies should assess the in vivo cytotoxicity of this pigment, determine its selective toxicity toward the target cells, and explore its cytotoxic mechanism.

## Materials and methods

### Fungal strains

A comprehensive study and assessment of pigment generation by twenty species of fungi that were isolated from rhizosphere soil samples of several plants (Fig, Lemon, Olive, Onion, and Sugarcane) in Qena Governorate, Egypt. The dilution plate method previously described by Waksman [21], with a slight modification, was employed to isolate the fungal flora associated with the collected rhizospheric soil samples. Briefly, 100 mL of sterilized distilled water was used to suspend 1 g of each soil sample. One milliliter of this suspension was transported to a sterilized petri dish, followed by 20 mL of sterilized potato dextrose agar (PDA) medium containing 200 g potatoes, 20 g dextrose, and 15 g agar in one liter of distilled water supplemented with 0.1 g chloramphenicol to suppress the bacterial growth. The plates were incubated at 28 °C for a week. The obtained colonies were identified morphologically and preserved on PDA slants.

### Pigment production and biomass assessment

To assess the pigment synthesis by the selected 20 rhizospheric fungal isolates, three distinct media were employed: Czapek-Dox broth (CzB), yeast extract dextrose peptone broth (YDPB), and potato dextrose broth (PDB; [22]. In distinct experiments, 50 mL of the designated media were each injected with one milliliter of spore suspension with a concentration of 1.5 × 10^8 spores/mL, produced from a fungal species culture that was 7 days old. The inoculation flasks were agitated at 160 rpm for 10 days at 30 °C [23]. Three experiments were performed.

### Extraction of pigments

Upon completion of the incubation period, the biomass and cell-free supernatant from the fungal isolates were harvested using centrifugation at 10,000 rpm and 4 °C for 10 min. The supernatant was evaporated under low pressure and subsequently lyophilized to get crude pigment powder utilizing a freeze dryer (VirTis: Model #6KBTES-55, Albany, NY, USA). 0.1 g of the crude pigment powder was dissolved in 20 mL of 95% ethyl alcohol, and the mixture was shaken at 180 rpm for 60 min on a shaker incubator [24]. The mycelial dry weight was evaluated as described by Velmurugan et al. [25]. *Aspergillus* sp. isolate Asmaa 2024 was found to be the potent isolate for pigment secretion, and the choice was dependent on the visual intensity of the extracted pigment.

### Characterization of the extracted pigments using UV‒Visible spectrophotometry

A UV-visible spectrophotometer was employed to assess the extracellular pigments across an extensive wavelength range (200–800 nm). The dilution factor was multiplied by the absorbance units (AU) recorded at the wavelength (ʎ) of peak absorption for each pigment to get the pigment concentration [25]. The obtained data confirmed the visual observation that *Aspergillus* sp. Asmaa 2024 had the highest activity. At 4 °C, it was kept on malt extract agar (MEA) slants, in addition to a cryopreserved sample at − 86 °C for further characterization experiments.

### Morphological characterization of *Aspergillus* sp. Asmaa 2024

In this investigation, *Aspergillus* sp. isolate Asmaa 2024 was morphologically recognized using various pertinent [26] criteria based on its macroscopic and microscopic characteristics. In a three-point design, 9-cm Petri plates containing Czapek’s agar (CZ), malt extract agar (MEA), and Czapek’s yeast extract agar (CYA) were inoculated with spore suspension from a seven-day-old culture [27]. After that, the plates were incubated for seven days at 25 °C in the dark. The MEA culture’s microscopic features were investigated.

### Molecular identification of the most active isolate

*Aspergillus* sp. isolate Asmaa 2024 DNA extraction was done using the procedure delineated by Moubasher et al. [28]. DNA isolation was carried out with the CTAB method, comprising 800 µL of a solution containing 3.0% CTAB, 1.4 M NaCl, 0.2% mercaptoethanol, 20 mM EDTA, 100 mM Tris-HCl at pH 8.0, and 1.0% PVP-40. The PCR protocol employed SolGent EF-Taq [29] alongside the universal primers ITS1 and ITS4 [30] to amplify the ITS region. The DNASTAR software (version 5.05) was utilized to produce a continuous sequencing of the *Aspergillus* sp. isolate Asmaa 2024. In the ITS dataset, eleven closely related strains of the *Aspergillus flavipes* group, including the type species, were obtained from GenBank among the thirteen species. Additionally, one sequence was associated with the *Aspergillus* species being studied, while another was ascribed to *A. fumigatus* ATCC 1022, serving as the outgroup. MAFFT was used to align every sequence [31] with default settings. The BMGE [32] was utilized to reduce the number of uninformative characters and alignment gaps. Phylogenetic analyses were performed with MEGA X version 10.2.6, employing maximum likelihood (ML) and maximum parsimony (MP) methods [33]. One thousand bootstrap replications were employed to evaluate the resilience of the most parsimonious trees [34]. The optimal nucleotide substitution model for maximum likelihood analyses was determined utilizing Model test 3.7’s implementation of the Akaike information criterion (AIC) [35]. The phylogenetic tree was created using MEGA X and then modified and saved as a TIFF file.

### Pigment characterization by FTIR analysis

FTIR was conducted at the Central Laboratory of South Valley University, Qena, Egypt, using a Nicolet Magna-FTIR 560 spectrometer (USA). The functional groups were detected at a wavenumber range of 4000–400 cm^− 1^ by mixing 10 mg of *Aspergillus* sp. isolate Asmaa 2024 pigmented secondary metabolites residue with 100 mg of KBr powder, following the method described by Li et al. [36]

### GC‒MS analysis

The ethanol extract of the *Aspergillus* sp. strain Asmaa 2024 pigment was analyzed using a GC-TSQ mass spectrometer (Thermo Scientific, Austin, TX, USA) at Nawah Scientific Center, Mokattam, Egypt. The studies used a capillary column TG-5MS (30 m x 0.25 mm x 0.25 μm film thickness) with Helium as the carrier gas at a steady flow rate of 1 ml/min. The temperature of the column oven was first maintained at 60 °C, then raised by 5 °C/min to 250 °C with a 2-minute hold, and finally raised to 300 °C at a rate of 30 °C/min. The solvent delay was 4 min, and diluted samples of 1 µL were injected automatically using Autosampler AS3000 coupled with GC in the split mode. EI mass spectra were collected at 70 eV ionization voltages over the range of m/z 50–650 in full scan mode. The ion source and transfer line were set at 200 °C and 280 °C, respectively. The components were identified by comparison of their mass spectra with those of WILEY 09 and NIST14 mass spectral databases [37].

### Elemental analysis

A Thermo Scientific FLASH 2000 Organic Elements Analyzer (Thermo Fisher Scientific, USA) was used by the Analytical Chemistry Unit (ACAL) at Assiut University in Egypt to ascertain the fundamental components C, H, N, O, and S of the pigment from the *Aspergillus* sp. isolate Asmaa 2024.

### EDX and SEM analysis

The extracted pigment was subjected to EDX spectroscopy to determine the elemental contents. After applying a thin coating of pigment residue to the adhesive carbon tape that was affixed to the aluminum stub, the tape was gold-coated for 3 min. Then the elemental composition and the particle morphology were analyzed using Burker NanoBerlin, Germany [1].

### Antibacterial efficacy of the crude pigment

Bacterial strains used in this study were provided by the International Luxor Hospital and the bacteriology laboratory at the Faculty of Science at South Valley University (Table 1). A 0.2 μm-syringe filter was used to sterilize the solution after diluting 0.5 g of crude pigment in 10 mL of distilled water for the test, so the concentration of the stock pigment solution was 50 mg per mL. The antibacterial properties of the crude pigment as minimum inhibitory concentration (MIC) and minimum bactericidal concentration (MBC) were conducted against various bacteria, including *Escherichia coli* BLSVU1, *Klebsiella pneumoniae* BLSVU2, *Proteus vulgaris* BLSVU3, *Pseudomonas cepacia* BLSVU4, *Pseudomonas fragi* BLSVU5, *Enterobacter cloacae* BLSVU6, *Enterobacter aerogenes* BLSVU7, *Serratia liquifaciens* BLSVU8, *Staphylococcus aureus* BLSVU9, *Staphylococcus epidermidis* BLSVU10, *Streptococcus pyrogenes* BLSVU11, *Bacillus subtilis* BLSVU12, and *Enterococcus faecalis* BLSVU13 by using broth-dilution microtiter technique and INT (p-iodonitrotetrazolium violet chloride) formazan assay in 96 well plates. A 24-hour aerobic growth on tryptic soy agar (TSA) at 37 °C was used to prepare the inoculum. In a 96-well microtiter plate, a concentration of 0.001 OD595 was achieved by combining 100 µL of tryptic soy broth (TSB) with amounts ranging from 10 to 100 µL of the crude pigment to make concentrations ranging from 4.5 to 25 mg/mL in columns from 1 to 10 (8 replicates were performed). Column 11 is considered a negative control containing 100 µL of culture media without bacteria, and column 12 is a positive control containing 100 µL of culture media and bacteria without the fungal pigment. Chloramphenicol of 25 mg/mL has been used as another positive control. After that, the plates were placed in an aerobic incubator set at 37 °C for a duration of 24 h. Finding the lowest concentration at which the color of INT remained unchanged was defined as MIC [38]. After the MIC determination, aliquots of 50 µL from all wells, which showed no visible bacterial growth (preventing colour change of INT), were seeded in TSA (3 replicates) followed by aerobic incubation at 37 °C for 24 h. Now, any organisms that the MIC test suppressed but did not kill have an opportunity to proliferate because the fungal pigment has been diluted significantly. The lowest antibacterial agent concentration that has reduced the number of colonies by 99.9% following a typical incubation period serves as an indicator of MBC [15, 39, 40].

**Table 1:** Tested bacterial strains code, source, and sensitivity

Strain	Code	Source of isolation	Sensitivity
*Escherichia coli*	BLSVU1	Urine	Resistant to ampicillin-sulbactam, ampicillin, gentamicin, nalidixic acid, amikacin, ceftazidime, ciprofloxacin, piperacillin, piperacillin-tazobactam, and cefepime, while sensitive to meropenem and Imipenem.
*Klebsiella pneumoniae*	BLSVU2	Blood	Resistant to ampicillin-sulbactam, ampicillin, meropenem, and Imipenem, while sensitive to colistin and amikacin.
*Proteus vulgaris*	BLSVU3	Urine	Resistant to ampicillin-sulbactam, ampicillin, gentamicin, nalidixic acid, amikacin, ceftazidime, ciprofloxacin, piperacillin, piperacillin-tazobactam, cefepime, while sensitive to meropenem and Imipenem.
*Pseudomonas cepacia*	BLSVU4	Fish	Sensitive: Tetracycline, Chloramphenicol, Nalidixic acid, Aztreonam, Sulfamethoxazole-trimethoprim, Norfloxacin, and Gentamycin, while resistant to erythromycin and penicillin G.
*Pseudomonas fragi*	BLSVU5	Fish	Sensitive: Tetracycline, Chloramphenicol, Sulfamethoxazole-trimethoprim, and Norfloxacin, while resistant to erythromycin and penicillin G.
*Enterobacter cloacae*	BLSVU6	Surgery rooms	Sensitive: meropenem, gentamicin, ciprofloxacin, Resistant: cefixime, ceftazidime, cefepime, and cefpirome.
*Enterobacter aerogenes*	BLSVU7	Surgery rooms	Sensitive: imipenem, amikacin, ciprofloxacin. Resistant: cefixime, ceftazidime, cefepime, and cefpirome
*Serratia liquifaciens*	BLSVU8	Fish	Sensitive: Tetracycline, Chloramphenicol, Sulfamethoxazole-trimethoprim and Norfloxacin, while resistant oxacillin, cefaclor, cefazolin,
*Staphylococcus aureus*	BLSVU9	Urine	Sensitive: nitrofurantoin, vancomycin, clindamycin and trimethoprim-sulfamethoxazole, while resistant to penicillin, erythromycin, and tetracycline.
*Staphylococcus epidermidis*	BLSVU10	Fish	Sensitive: Tetracycline, Chloramphenicol, Sulfamethoxazole-trimethoprim Gentamycin, Norfloxacin, Vancomycin, Rifampicin, Ceftriaxone and Clindamycin while resistant to ampicillin, erythromycin, streptomycin, and amoxicillin.
*Streptococcus pyrogenes*	BLSVU11	Blood	Sensitive to penicillin, amoxicillin and ceftriaxone, while resistant to clindamycin and Tetracycline.
*Bacillus subtilis*	BLSVU12	Soil	Sensitive to gentamicin and ciprofloxacin, while resistant to cefotaxime and ampicillin
*Enterococcus faecalis*	BLSVU13	Fish	Sensitive: Tetracycline, Chloramphenicol, Sulfamethoxazole-trimethoprim, Gentamycin and Norfloxacin, while resistant to ampicillin, erythromycin.

### Static biofilm formation and antibiofilm efficacy of the crude pigment

The different bacterial strains’ spore suspensions were made in TSB medium with an optical density (OD 595) of 0.02. The bacterial solution was added to 130 µL of each well of a 96-well microtiter plate (U Bottom, Sterilin). A subsequent 24 h were spent incubating the plate at 37 °C. The wells were rinsed six times with distilled water after incubation. After that, the biofilm was left to sit in 160 µL of 96% ethanol with a 0.1% crystal violet solution for 10 min [40–44]. Controls included wells containing medium and wells containing only ethanol, devoid of fungal pigment. After that, distilled water was used to rinse the plate four more times. Each empty well had 210 µL of 96% ethanol added to it in order to eliminate the crystal violet stain that had been retained by the biofilm. Afterwards, 50 µL of the unprocessed pigment was added to every well, and the plate was then kept in an incubator at 37 °C for an additional day. Using a microplate reader (Infinite^®^ F50 Ostrich), the absorbance was measured at 595 nm [41, 42]. Equation (1) was used to determine the % biofilm inhibition.

1 $$\bf \% {\text{ Biofilm inhibition }} = {1}00 - \frac{{{\mathrm{Abs}}.{\text{ of Sample}} - {\mathrm{Abs}}.{\text{ Blank}}}}{{{\mathrm{Abs}}.{\text{ Control }} - {\text{ Abs}}.{\text{ Blank}}}} \times {1}00 $$

where Abs. of sample is the absorbance of treated bacterial strain with fungal pigment at 595 nm; Abs. of blank is the absorbance of culture media without tested bacteria and fungal pigment; Abs of control is the absorbance of tested bacterial strain without fungal pigment.

### Particle size and zeta potential measurements

The fungal pigment’s size distribution was examined using a photon correlation spectroscopy system set at a fixed angle of 173° and a temperature of 25 °C. The analyzer, a Zetasizer Nano ZN from Malvern Panalytical Ltd., UK, was used to determine the particle size. The analysis focused on the average volume diameter and polydispersity index; measurements were triplicated. The system solvent was water, and sample were diluted to reach about 10^8^ particles/ ml.

### Determination of the fungal pigment’ antibacterial efficacy by SEM spectroscopy

The *K. pneumoniae* thin films were incubated in a 4% glutaraldehyde solution in a 0.05 M phosphate buffer (pH 7.0) at 4 °C for a duration of 12 h. After that, we used a series of graded ethanol to dehydrate the slides, and then we used critical point drying equipment using liquid carbon dioxide to dry them according to Wang et al. [45]. The samples underwent three washes in phosphate buffer before being coated with gold and examined under a Japanese microscope (JEOL JSM-5500LV).

### Cytotoxic properties of the fungal pigment

The cytotoxic effectiveness of the fungal pigment was in vitro evaluated on three distinct human tumor cell lines: osteosarcoma (HOS), lung carcinoma (A549), and skin cancer (A431). The Bioassay-Cell Culture Laboratory managed and established the procedures at Egypt’s National Research Centre in Cairo, 12622. The technique described by Alley et al. [46] was used to assess cell viability, and MTT (3-(4,5-dimethylthiazol-2-yl)-2,5-diphenyl tetrazolium bromide) was used as the indicator. The sample’s concentration range (100–0.78 µgmL) was utilized to ascertain the rate of mitochondrial degradation of MTT to purple formazan. A suspension of selected tumor cell lines was prepared using a combination of RPMI 1640 medium, a 1.0% antibiotic-antimycotic solution (10,000 U/mL potassium penicillin, 10,000 µg/mL streptomycin sulfate, and 25 µg/mL amphotericin B), and 1% l-glutamine. The suspension was kept at 37 °C in a 5% CO_2_ environment. The cells were cultivated in clusters after ten days. They were then inoculated with fresh growth media at a density of 10 × 103 cells/well into 96-well microtiter plates. In a water-jacketed carbon dioxide incubator (Sheldon, TC2323, Cornelius, OR, USA) with less than 5% CO_2_, the plates were incubated for 24 h at 37 °C. Following the elimination of active media, serum-free replacement media were developed. Incubation was conducted on cells subjected to various doses of the fungal pigment, alongside cells devoid of these substances (negative control). After 48 h of incubation, each well received 40 µL of MTT salt (2.5 µg/mL), followed by an additional 4 h of incubation at 37 °C in a 5% CO_2_ atmosphere. The reaction was concluded by introducing 200 µL of 10% sodium dodecyl sulfate (SDS) to facilitate the dissolving of the resultant crystals, followed by overnight incubation at 37 °C. In identical experimental circumstances, a positive control of 100 µg/mL *Annona cherimolia* extract led to complete cell mortality [47]. The absorbance of the final product was quantified at 595 nm, with 630 nm serving as a reference, using a multi-well microplate reader following dissolution in 0.2% DMSO. Through probit analysis in SPSS 11, the lethal doses that resulted in 50% and 90% cell mortality within 48 h were estimated. We calculated the percentage change in cell viability from the following equation [Reading of treatment/Reading of negative control) -1] x 100.

### Statistical analysis

Three distinct studies were conducted to assess the variability in the data, expressed as means ± standard deviations (means ± S.D.). The data were compared using the LSD test and statistically evaluated by one-way ANOVA. This comparison was conducted between the control and the treatment groups utilizing the SPSS 16 software, with significance determined at the *p* < 0.05 level.

## Results

### Pigment production

**Fig. 1:**
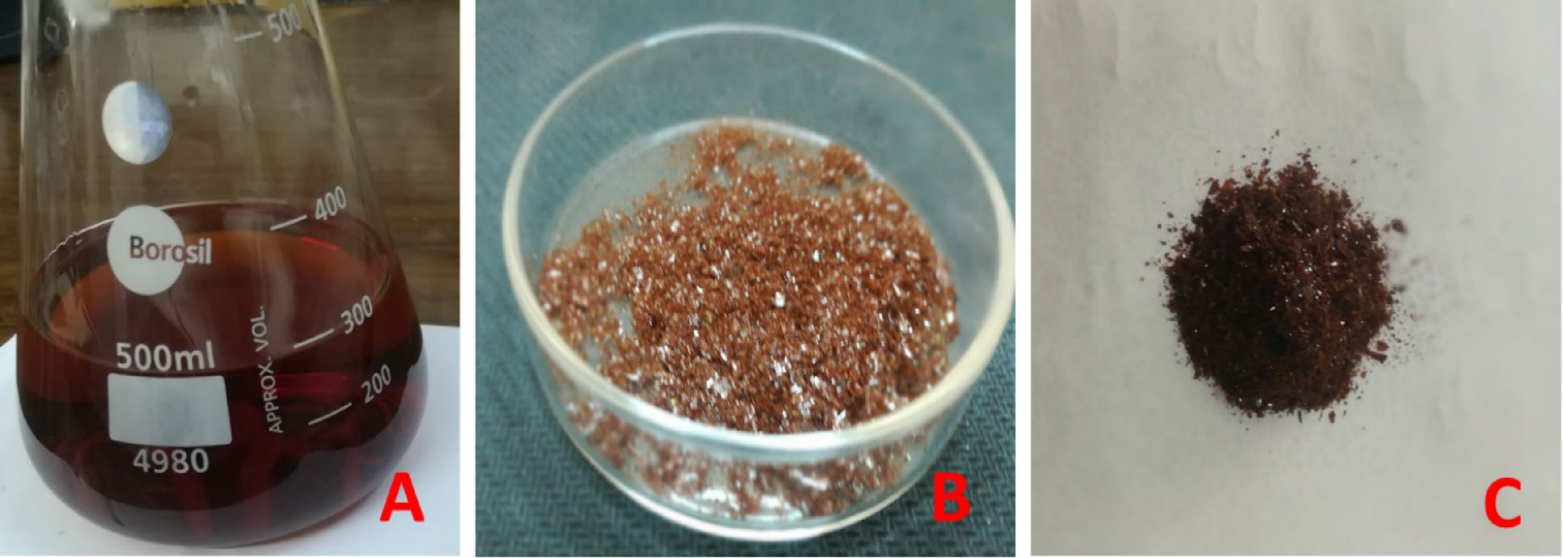
*Aspergillus frequens* strain Asmaa 2024 extracted pigment **A**: pigmentation on PDB medium and (**B**, **C**) pigment residue after lyophilization

The results indicated that ten isolates (50% of the total) were identified as pigment producers, and PDB was the most productive medium for pigment secretion, followed by CzB, whilst YDPB was the least effective. Nine isolates were capable of pigment development on PDB, whereas 7 isolates were pigment producers on CzB, and the produced concentrations were relatively low when compared with the other two used media. Six fungal isolates were able to synthesize pigmented secondary metabolites on YDPB submerged fermentation medium. Maximum pigment absorption occurred at wavelengths ranging from 380 to 500 nm, except for *Trichoderma viride*, which exhibited the maximum absorbance at 700 nm. *Aspergillus flavus*, *A. frequens*, *A. nidulans*, *A. ochraceus*, *A. sydowii*, *A. terreus*, *A. ustus*, *Stachybotrys elegans*, and *Trichoderma viride* exhibited pigment production on PDB. *A. frequens* exhibited the highest concentration (21.36 ± 1.8 AU/mL) of a reddish-brown pigment at 500 nm (Table 2; Fig. 1). *A. nidulans* filtrate ranked second, with a dark red hue displaying a concentration of 21.0 ± 0 AU/mL. *A. flavus*, *A. ochraceus*, and *A. terreus* released substantial quantities of colored secondary metabolites at 380 nm (Table 2).

**Table 2:** Pigment concentration (AU/mL) and fungal biomass (g/50 mL) of the tested fungi on PDB, YEDP, and CzB media

Fungal strains	PDB			YDPB			CzB		
	Pigment concentration (AU/mL)	ʎ_max_ (nm)	Biomass (g/50 mL)	Pigment concentration (AU/mL)	ʎ_max_ (nm)	Biomass (g/50 mL)	Pigment concentration (AU/mL)	ʎ_max_ (nm)	Biomass (g/50 mL)
*Aspergillus flavus*	2.2*±0.1	380	0.47 ± 0.08	–	–	0.9 ± 0.65	0.45*±0.05	380	0.6 ± 0.15
*A. frequens*	21.36*±1.8	500	0.95 ± 0.32	1.13*±0.13	380	0.97 ± 0.2	0.62*±0.16	380	0.43 ± 0.36
*A. nidulans*	21.0*±1.6	500	0.96 ± 0.3	1.5*±0.46	380	1.0 ± 0.2	–	–	0.53 ± 0.34
*A. ochraceus*	4.25*±0.22	380	0.63 ± 0.16	9.8*±0.5	380	0.58 ± 0.44	0.34*±0.054	380	0.45 ± 0.3
*A. sydowii*	1.25*±0.78	380	1.53 ± 0.4	–	–	0.5 ± 0.4	–	–	0.7 ± 0.3
*A. terreus*	3.57*±0.43	380	0.65 ± 0.13	7.0*±0.64	380	0.76 ± 0.1	1.5*±0.06	380	0.84 ± 0.26
*A. ustus*	1.42*±0.12	450	0.5 ± 0.32	6.4*±1.0	380	0.54 ± 0.17	7.0*±0.55	380	0.6 ± 0.3
*Curvularia lunata*	–	–	1.25 ± 0.46	–	–	1.53 ± 0.76	–	–	0.7 ± 0.13
*Fusarium dimerum*	–	–	0.46 ± 0.54	–	–	0.65 ± 0.25	–	–	0.43 ± 0.2
*F. oxysporum*	–	–	0.54 ± 0.15	–	–	0.5 ± 0.26	–	–	0.46 ± 0.24
*F. semitectaum*	–	–	0.4 ± 0.096	–	–	0.36 ± 0.26	–	–	0.4 ± 0.17
*F. solani*	–	–	0.36 ± 0.2	–	–	0.32 ± 0.07	–	–	0.22 ± 0.36
*Penicillium chrysogenum*	–	–	0.58 ± 0.0.1	–	–	0.56 ± 0.13	0.8*±0.06	380	0.6 ± 0.9
*P. duclauxii*	–	–	0.76 ± 0.46	–	–	0.67 ± 0.27	–	380	0.5 ± 0.15
*Stachybotrys elegans*	1.7*±0.09	450	0.97 ± 0.36	–	–	0.77 ± 0.27	1.07*±0.13	380	0.5 ± 0.2
*Scopulariopsis brevicaulis*	–	–	0.8 ± 0.4	–	–	0.54 ± 0.16	–	–	0.7 ± 0.34
*S. asperula*	–	–	0.62 ± 0.1	–	–	0.4 ± 0.2	–	–	0.66 ± 0.17
*Thermoascus aurantiacus*	–	–	0.7 ± 0.36	–	–	0.5 ± 0.2	–	–	0.4 ± 0.12
*Trichoderma viride*	0.84*±0.14	700	0.52 ± 0.1	0.15 ± 0.53	700	0.72 ± 0.2	–	–	0.23 ± 0.3
*Verticillium alboatrum*	–	–	0.86 ± 0.146	–	–	0.7 ± 0.1	–	–	0.56 ± 0.3

PDB: Potato dextrose broth; YDPB: Yeast extract dextrose peptone broth; CzB: Czapek’s broth

As illustrated in Table 2, the highest absorbance reading on CzB was observed in *A. ustus* (7 ± 0.55 AU/mL). Also, *A. terreus* and *S. elegans* produce significant levels of pigmented secondary metabolites, recording 1.5 ± 0.06 and 1.07 ± 0.13 AU/mL, respectively. Interestingly, CzB medium was the only medium that induced pigment secretion by *P*. *chrysogenum* with pigment intensity (0.8 ± 0.06 AU/mL). The maximum readings of the fungal-pigmented secondary metabolites were detected at a wavelength of 380 nm.

On YDPB, *A. ochraceus* produced the highest amount of pigment, 9.8 ± 0.5 AU/mL at wavelength 380 nm. *A. terreus* and *A. ustus* also recorded high readings at the same wavelength 380 nm (Table 2). *Trichoderma viride* recorded a non-significant reading (0.15 ± 0.53 AU/mL) at wavelength 700 nm (Table 2).

### Biomass estimation

The mycelial growth rates on PDB, YDPB, and CzB varied. Out of the isolates examined, 10 exhibited enhanced mycelial growth on PDB, 6 on YDPB, and 4 on CzB as shown in Table 2. The maximal mycelial dry weight (1.53 ± 0.76 g/50 mL) was recorded for *Curvularia lunata* on YDPB. On the other hand, *A. sydowii* had the highest mycelial dry weight on PDB at 1.53 ± 0.4 g/50 mL, succeeded by *C. lunata* with a mycelial dry weight of 1.25 ± 0.46 g/50 mL. The mycelial dry weights of *A. nidulans* on PDB and YDPB media were 1.0 ± 0.216 g/50 mL and 0.96 ± 0.3 g/50 mL, respectively, suggesting that the growth of *A. nidulans* on PDB was comparable to that on YDPB. Similarly, the growth results for YDPB and PDB were comparable for the proficient pigment-producing strain *A. frequens*, yielding mycelial dry weights of 0.97 ± 0.2 and 0.95 ± 0.32 g/50 mL, respectively. *A. ustus* exhibited the most significant mycelial development on CzB, recorded at 0.84 ± 0.26 g/50 mL (Table 2).

### Morphological identification of *Aspergillus* sp. Asmaa 2024

**Fig. 2:**
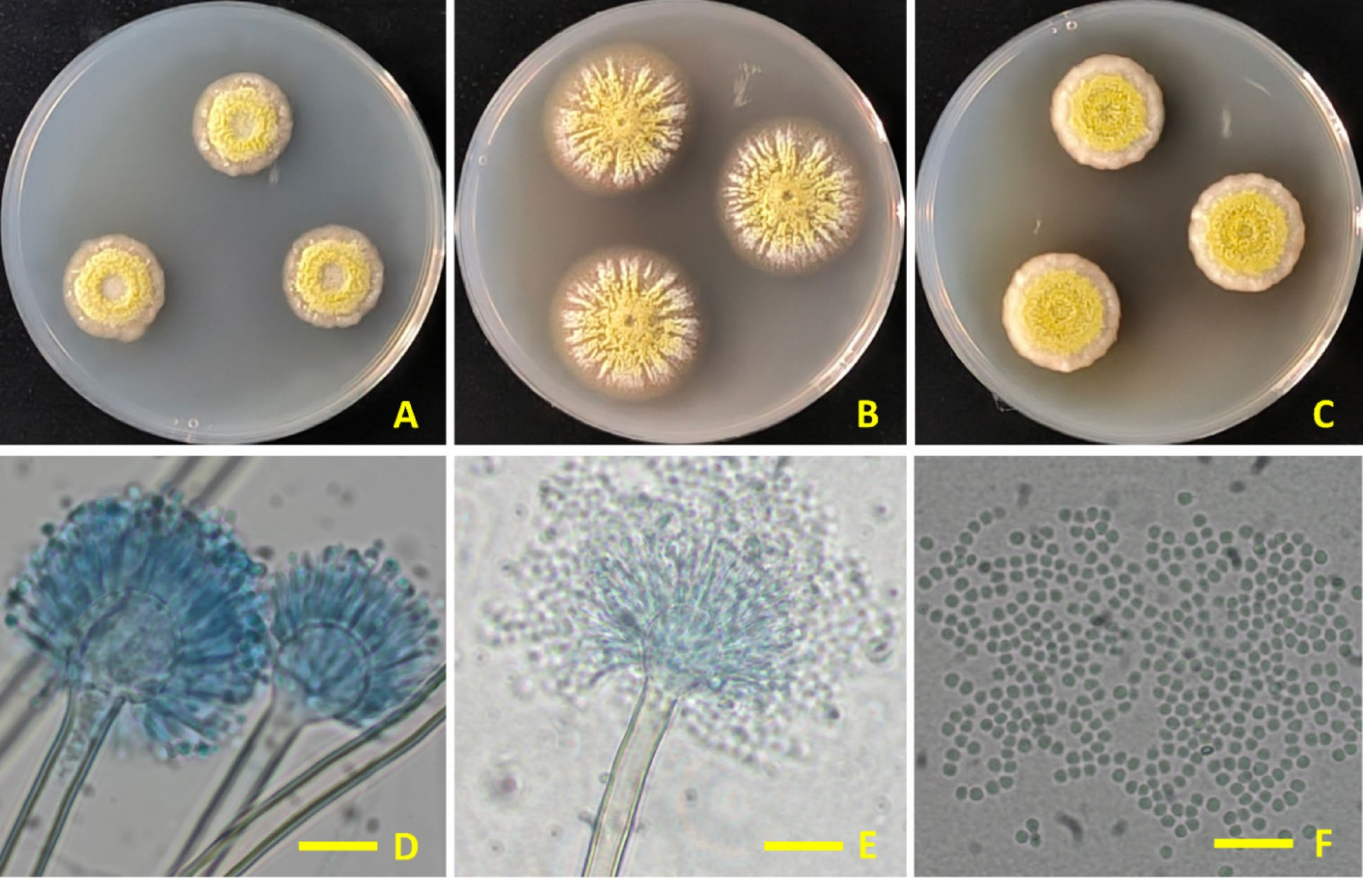
*Aspergillus frequens* strain Asmaa 2024 (**A**–**C**) seven-day-old colonies on Cz, MEA, and CYA at 25 °C, respectively. (**D**–**E**) Conidiophores and conidial heads (**F**) Conidia (Scale bar = 10 μm)

Morphologically, the *Aspergillus* sp. isolate Asmaa 2024 in this study exhibited identical features to the type material of *Aspergillus frequens*. Colonies measured 30, 45, and 35 mm in diameter after 7 days at 25 °C on Cz, MEA, and CYA, respectively. Conidial heads are loosely radiate to radiate, with biseriate conidiophores exceeding 1000 μm in length. Vesicles are primarily pyriform or subglobose, measuring 5.0–15 μm in diameter. Metulae measure 4–6 μm in length. Phialides measuring 4–6 μm. Conidia are globose to subglobose, measuring 2.5–3 μm (Fig. 2).

### Molecular identification of the most active strain

**Fig. 3:**
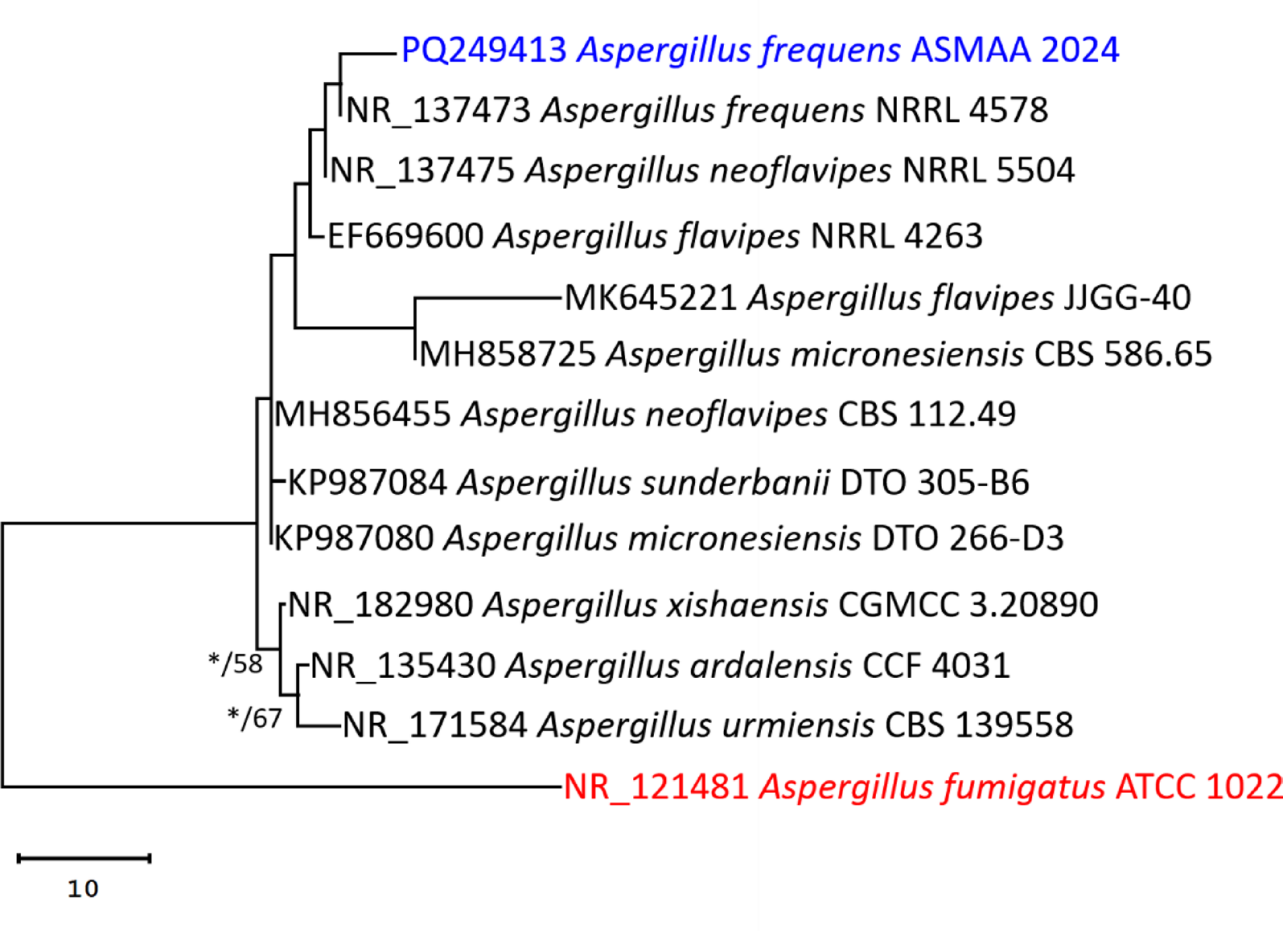
Maximum parsimoneous phylogenetic tree produced by ML/MP analysis of *A. frequens* strain Asmaa 2024’ ITS sequence in this work in comparison to sequences of the *A. flavipes* group’s most closely related species in GenBank. Near the corresponding nodes are the bootstraps (1000 replications) with ML/MP support values ≥ 50%. *A. fumigatus* ATCC 1022 was used as the tree root

The maximum parsimony dataset consisted of 618 characters, including 117 constant characters (devoid of gaps or N characters), 20 variable and parsimony-uninformative characters (17.1% of the constant characters), and 3 parsimony-informative characters (2.6% of the constant characters). The most suitable model for nucleotide substitution was the Tamura 3-parameter model (T92). The results of choosing the most parsimonious tree (103 steps, final maximum likelihood optimization value of − 1350.98, consistency index of 0.8, retention index of 0.75, and composite index of 0.6) to clarify and examine the evolutionary relationships across taxa was shown in Fig. 3. The evolutionary data illustrated the species’ affiliations with *Aspergillus flavipes* group. The phylogenetic analysis revealed that strain Asmaa 2024 of the *Aspergillus* species is phylogenetically associated with the type material, *A. frequens* NRRL 4578, within the same clade. So, the strain was designated as *A. frequens* (Fig. 3).

### FTIR analysis of *A. frequens* pigment

**Fig. 4:**
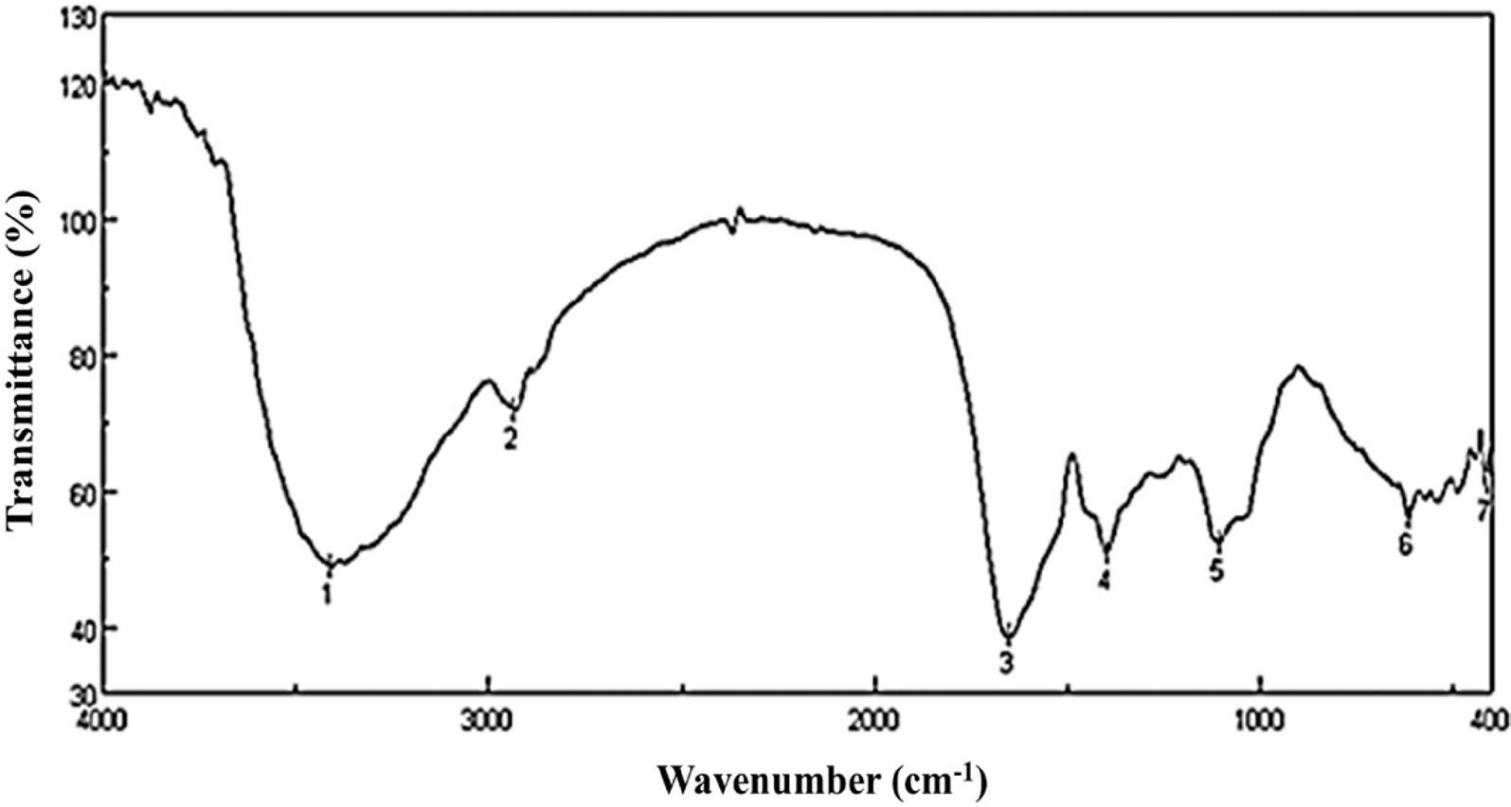
FTIR analysis of the crude pigment produced by *A. frequens*

The spectra exhibited prominent absorption bands at 3411, 2935, 1652, 1399, 1106, 616, and 415 cm^− 1^, wavenumbers (Fig. 4). The broad peak appears at 3411 cm^− 1,^ corresponding to the O-H stretching group of alcohols. The observed peak at 2935 cm^− 1^ reflects the presence of alkanes and aliphatic hydrocarbons in the side chain of the extracted fungal pigment. The detected sharp peak at a wavenumber of 1652 cm^− 1^ was due to alkenes (C = C) and C = O of ketones and quinones. The presence of C-N or CH_3_ bending was confirmed by the observed peak at 1399 cm^− 1^, suggesting the presence of aliphatic or aromatic amines or amino acid derivatives, or methylated organic compounds. The peak shown at wavenumber 1106 cm^− 1^ reflects the presence of C-O-C and C-N stretching of ether-ester linkages and C-N stretching of nitrogen-containing compounds, indicating the presence of oxygenated compounds such as pyrone. The detected peaks in the fingerprint region at 616 and 415 cm^− 1^ correspond to the presence of substitution and deformation in the aromatic rings. These findings suggest the presence of phenolic, amine, and aromatic structures in the extracted pigment.

### GC‒MS analysis of *A. frequens* extracellular pigment

**Fig. 5:**
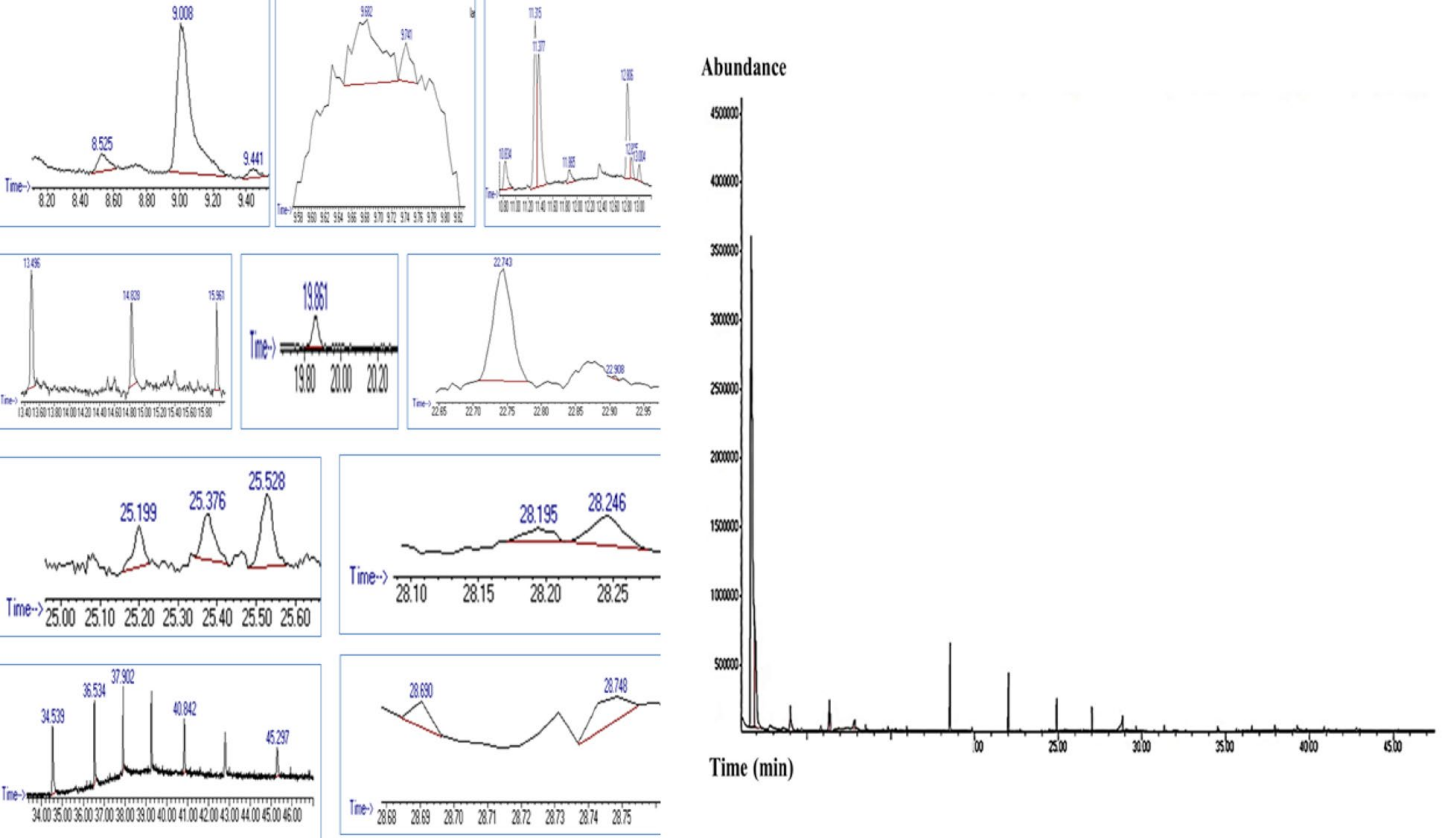
GC-MS chromatogram of *A. frequens* pigmented secondary metabolites

An analysis of the extracellular *A. frequens* pigmented secondary metabolites was conducted via GC‒MS. The results presented in Table 3; Fig. 5 revealed the presence of 31 bioactive compounds. Several colored compounds have been discovered, such as 2,6-Dimethyl-γ-Pyrone (pale yellow), 3-(2-Aminophenyl) Isocoumarin, a polyketide pigment with a yellow-orange color. Other chromophores, including 9-Acetyl-14-ethyl-13,14-dihydro-21-(methoxyca rbonyl)-43-Phorbinepropanoic acid, 3-(4-Chlorophenyl)-4,6-Dimethoxy-1-(prop-2’-e nyl)Indole-7-Carbaldehyde, and N-ethyl-1,3-dithioisoindoline;1 H-Isoindole-1,3(2 H)-dithione, 2-ethyl. The other detected auxochromes compounds, 6-Aza-5,7,12,14-tetrathiapentacene (sulfur-rich compound) and ((Z, Z)-1 H-Pyrrole-3-Propanoic Acid, 5-[(4-Ethenyl-1,5-Dihydro-3-Methyl-5-Oxo-2 h-Pyrrol-2-Ylidene)Methyl]-2-[[5-(Methoxycarbonyl)-3-(3-Methoxy-3-Oxopropyl)-4-Methyl-2 h-Pyrrol-2-Ylidene]Methyl]-4-Methyl-, Methyl Ester)), alongside azaphilone acyl donors fatty acid, hexadecanoic acid. Notably, two amino acids, l-Alanyl-l-alanyl-l-alanine methyl ester and 2-methylpiperazine (Table 3; Fig. 5), were also detected in the GC-MS chromatogram, providing reactive amine groups capable of forming the characteristic azaphilone nitrogen conjugates by substituting oxygen in the pyrone ring with nitrogen and subsequently altering the orange azaphilone to red ones.

**Table 3:** Identified compounds in *A. frequens* pigmented secondary metabolites by GC-MS analysis

Identified compound	Retention time (RT)	Area%	Chemical formula	Molecular weight (g/mol.)	Biological activities
9-Acetyl-14-ethyl-13,14-dihydro-21-(methoxycarbonyl)-43-Phorbinepropanoic acid	9.683 min	0.410%	C_55_H_76_N_4_O_6_	905.2	–
Monomethyltaurine	25.199 min	0.039%	C_3_H_9_NO_3_S	139.18	Antioxidant [67]
S-(2-Amino-2-carboxyethyl)- DL-Homocysteine,	11.881 min	0.887%	C_7_H_14_N_2_O_4_S	222.26	Antioxidants Waters et al. [68]
((Z, Z)-1 H-Pyrrole-3-Propanoic Acid, 5-[(4-Ethenyl-1,5-Dihydro-3-Methyl-5-Oxo-2 h-Pyrrol-2-Ylidene)Methyl]-2-[[5-(Methoxycarbonyl)-3-(3-Methoxy-3-Oxopropyl)-4-Methyl-2 h-Pyrrol-2-Ylidene]Methyl]-4-Methyl-, Methyl Ester)	37.905 min	0.171%	C_33_H_36_N_4_O_6_	584.66	Antimicrobial, anticancer, and anti-inflammatory properties [69]
2,6-Dimethyl-Gamma.-Pyrone	29.64 min	0.631%	C_7_H_8_O	124.14	I key chemical intermediate in the synthesis of pharmaceuticals, dyes, and polymers [70]
3-(2-Aminophenyl)Isocoumarin	13.005 min	0.387%	C_15_H_11_NO_2_	237.25	Anti-cancer, anti-inflammatory, anti-bacterial, and anti-fungal agents [71]
l-Alanyl-l-alanyl-l-alanine methyl ester	28.201 min	0.032%	C_7_H_14_N_2_O_3_	174.2	Intermediate in creating anticancer and anti-HIV drugs [72]
3-(4-Chlorophenyl)-4,6-Dimethoxy-1-(prop-2’-e nyl)Indole-7-Carbaldehyde	24.902 min	0.015%	C_20_H_18_ClNO_3_	355.81	Anticancer activity, anti-inflammatory and antimicrobial [73]
Butyraldehyde diethyl acetal	10.837 min	1.651%	C_8_H_18_O_2_	146.23	–
1,1-Diaethoxy-Aethan	6.687 min	80.884%	C_6_H_14_O_2_	118.17	–
1,1, 3-Triethoxypropane	14.83 min	2.775%	C_11_H_24_O_4_	220.31	–
2-Ethyl Butyric acid	9.013 min	3.060%	C_5_H_10_O_2_	102.13	–
2-Methylpiperazine	25.531 min	0.770%	C_5_H_12_N_2_	100.16	Anti-inflammatory and antiviral Liu et al. [74]
3-(1-Propylbutylidene)-Carbazic acid, ethyl ester	8.529 min	0.554%	C_7_H_14_O_3_	146.18	–
3,16Bis[(trimethylsilyl)oxy]pregn-5-en-20-one o-methyloxime	22.745 min	1.015%	C_28_H_51_NO_3_Si_2_	505.8804	–
3-Piperidinol	29.075 min	0.405%	C_5_H_11_NO	101.15	Anti-tuberculosis, anti-inflammatory, anticonvulsant [75]
4-Amino-1-pentanol	19.866 min	0.119%	C_5_H_13_NO	103.16	A precursor or component in the synthesis of other antimicrobial agents and anticancer compounds [76, 77]
4-Fluoro-3-[1-hydroxy-2-(methylamino)ethyl]ph enol	28.69 min	1.081%	C_9_H_12_FNO_2_	185.198	–
6-Aza-5,7,12,14 tetrathiapentacene	15.961 min	0.522%	C_17_H_9_NS_4_	355.5	Showing activity against *Staphylococcus epidermidis* [78]
7,8-Didehydro-4,5-epoxy-17-methyl-3,6-bis[(tri methylsilyl)oxy]-, (5.alpha.,6.alpha.)- Morphinan	36.536 min	0.230%	C_23_H35NO3Si2	429.70	–
Acetic acid 2-Methylbutyl ester	11.315 min	0.434%	C_7_H_14_O_2_	130.1849	–
Cystine	25.38 min	0.074%	C_3_H_7_NO_2_S	121.16	–
Ethyl n-(2,5-dicarbomethoxyphenyl)Carbamate	13.501 min	0.122%	C_12_H_14_N_2_O_4_	250.25	synthesis of various thiazole compounds [79]
N-ethyl-1,3-dithioisoindoline; 1 H-Isoindole-1,3(2 H)-dithione, 2-ethyl	9.741 min	1.683%	C_10_H_13_NS_2_	207.309	–
1-(2,2-Diethoxyethyl)-5-methyl-1 H-Pyrimidine-2 ,4-dione	12.807 min	0.125%	C_11_H_18_N_2_O_4_	242.27	–
Trichloro Acetic acid	9.444 min	0.117%	C_2_HCl_3_O_2_	163.378	–
Thiocyanic acid, 5-alpha-cholestan-3beta-yl ester	40.843 min	0.526%	C27H47NS	429.74	–
Ursane-3,16-Diol	12.889 min	0.172%	C_30_H_50_O_2_	442.728	–
Hexadecanoic acid	28.591 min	0.608%	C_16_H_32_O_2_	256.43	–
Benzenepropanamine, N-(1,1-Dimethylethyl)-Alpha-Methyl-Gamma-Phenyl	45.302 min	0.502%	C_20_H_27_N	281.44	–

### Elemental analysis of *A. frequens*’ extracellular pigment

The C, H, N, O, and S elemental ratios of the crude pigment produced by *A. frequens* were as follows: Carbon (62.8263%), Hydrogen (7.813%), Nitrogen (5.688%), Oxygen (12.07%), and Sulfur (2.972%).

### SEM and EDX analysis of the extracted pigment

**Fig. 6:**
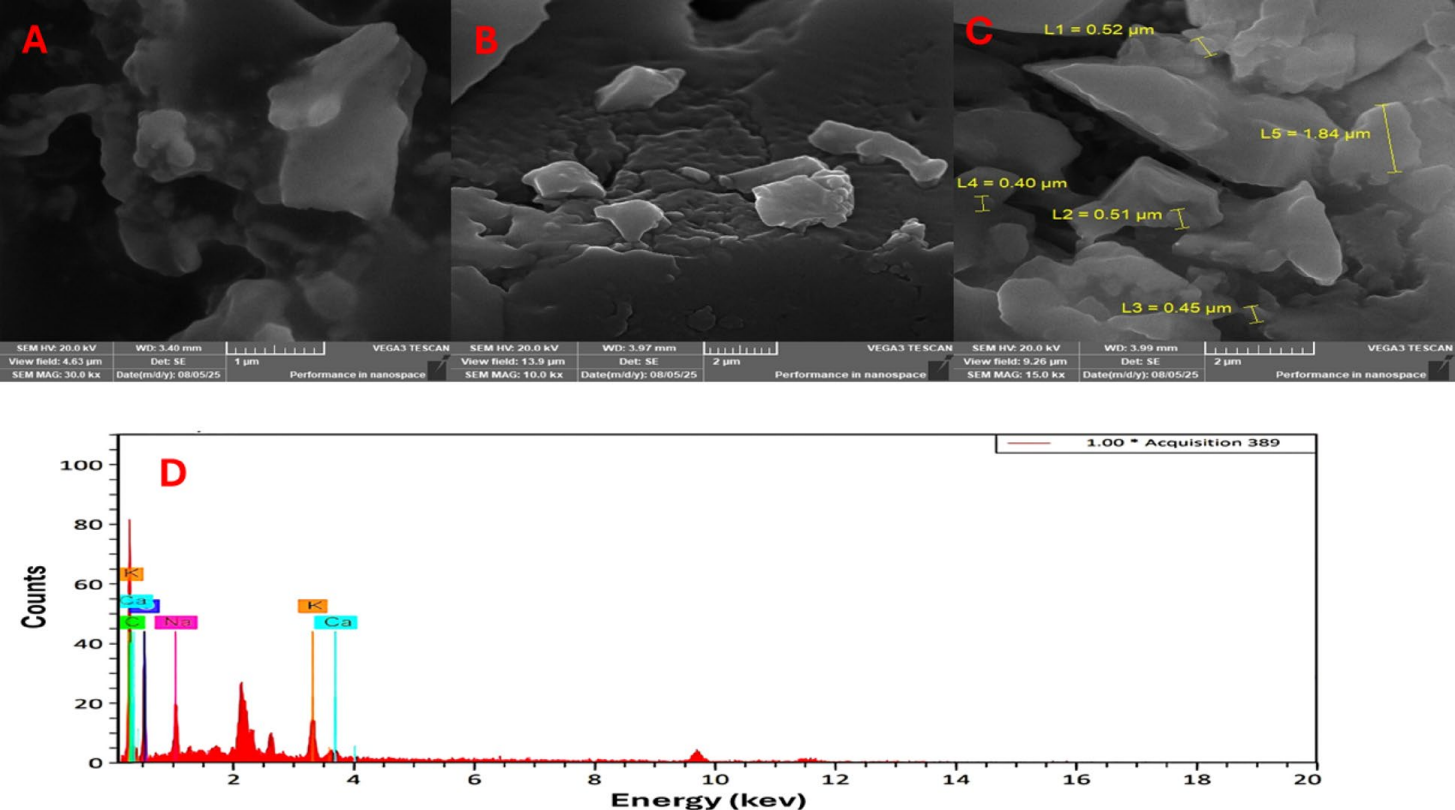
SEM micrographs (**A**, **B**, and **C**: showing particle size) at Scale bars = 1 and 2 μm and EDX analysis (**D**) of the extracted *A. frequens* pigment

SEM analysis proved to be an effective technique for examining the shape of the extracted pigment particles. As shown in Fig. 6A–C. the pigment exhibited a polymorphic nature, displaying irregular cubic and spherical forms with particle size range of 40–184 nm (Fig. 6C). EDX analysis further verified the predominance of carbon (57.52%) and oxygen (37.69%), confirming the pigment’s organic origin alongside detectable amounts of sodium (2.55%), potassium (1.76%), and calcium (0.47%), as presented in Fig. 6D.

### Antibacterial efficacy of *A. frequens*’ extracellular pigment

**Fig. 7:**
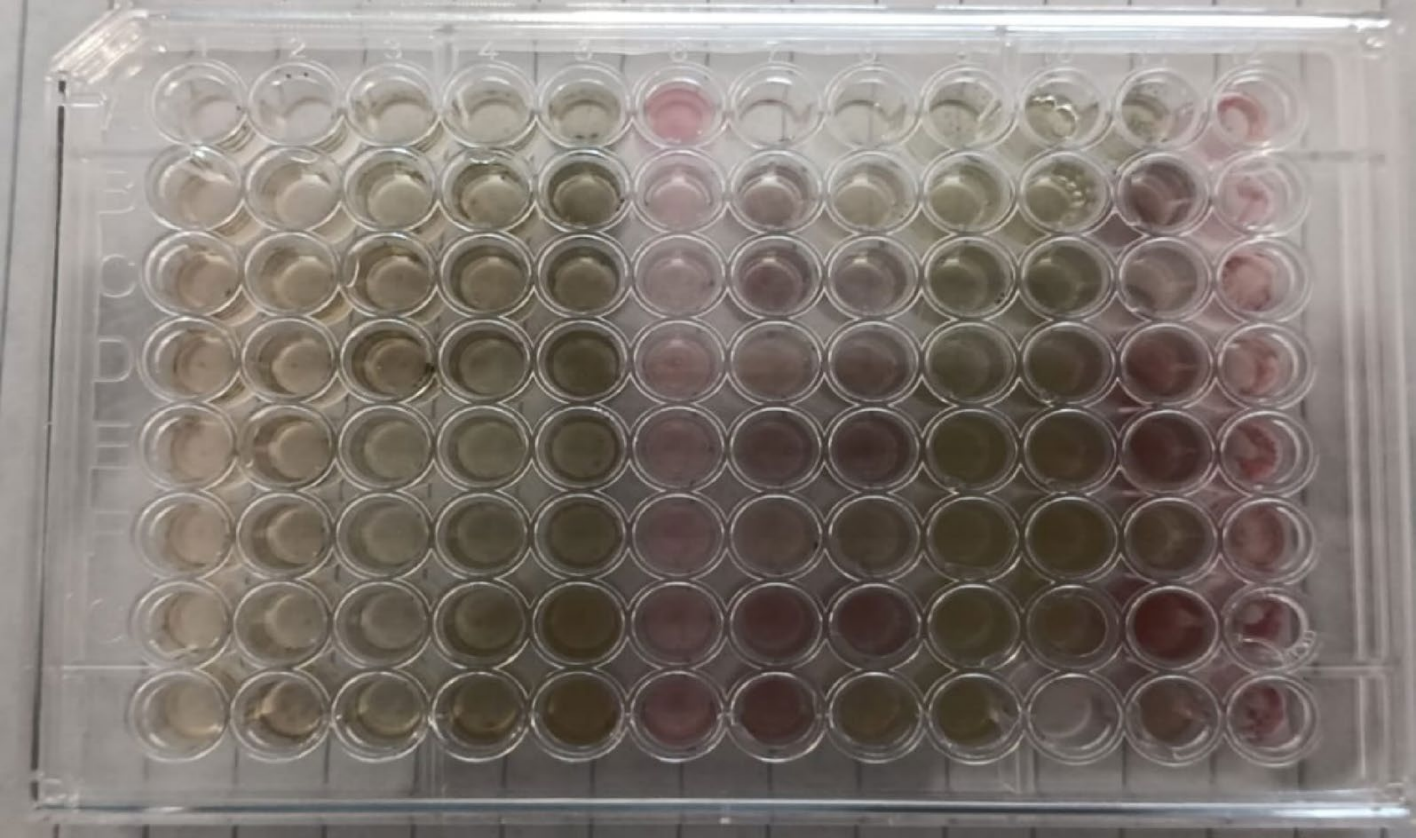
Antibacterial activity of fungal pigment against some pathogenic bacteria in a 96-well plate

For Gram-negative bacteria, the maximum MIC and MBC values (16.7 mg/mL) have been determined for *E. aerogenes* and *P. fragi*, while *K. pneumoniae*, *P. cepacia*, *S. liquefaciens*, and *E. cloacae* ranked second, with MIC and MBC values of 11.5 mg/mL. *E. coli* and *P. vulgaris*, conversely, attained the lowest result of 4.5 mg/mL. For Gram-positive bacteria, *S. pyrogenes*, *B. subtilis*, and *E*. *faecalis* recorded the highest MIC and MBC values of 16.7 mg/mL. At the same time, *S. aureus* and *S. epidermidis* came in second rank with MIC and MBC values of 11.5 mg/mL (Table 4; Fig. 7).

**Table 4:** Antibacterial activity (represented as MIC and MBC) of the crude pigment produced by *A. frequens* strain Asmaa 2024 against some pathogenic bacteria

Bacterial species		MIC (mg/mL)	MBC (mg/mL)
Gram -ve	*E. coli*	4.5	4.5
	*P. vulgaris*	4.5	4.5
	*K. pneumoniae*	11.5	11.5
	*P. cepacia*	11.5	11.5
	*S. liquifaciens*	11.5	11.5
	*E. cloace*	11.5	11.5
	*E. aerogenes*	16.7	16.7
	*P. fragi*	16.7	16.7
Gram + ve	*S. aureus*	11.5	11.5
	*S. epidermidis*	11.5	11.5
	*S. pyrogenes*	16.7	16.7
	*B. subtilis*	16.7	16.7
	*E. faecalis*	16.7	16.7

*: MIC: Minimum inhibitory concentration; MBC: Minimum bactericidal concentration

### Static biofilm formation and antibiofilm efficacy of *A. frequens*’ extracellular pigment

**Fig. 8:**
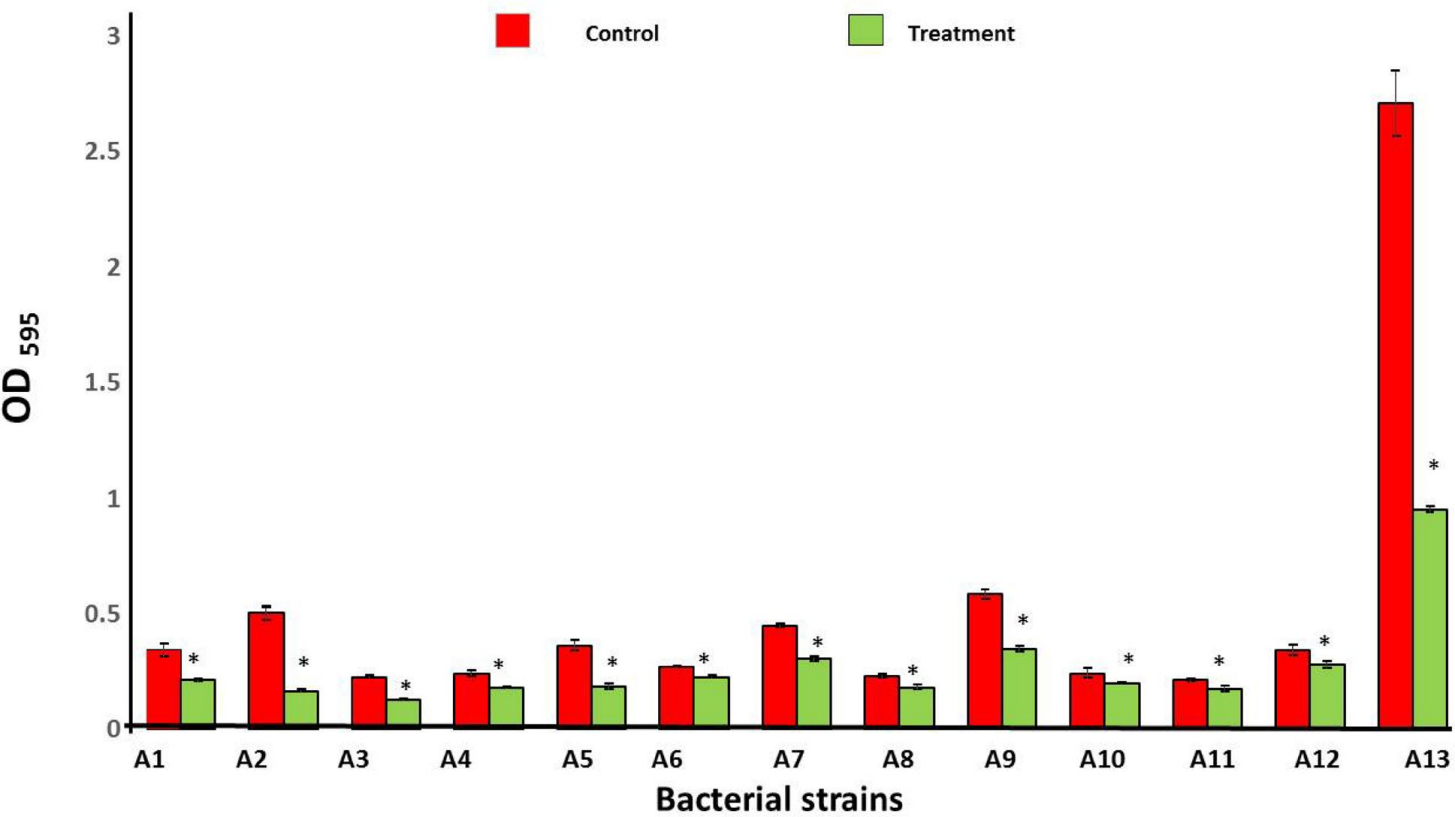
Antibiofilm activity of pigmented metabolites of *A. frequens* strain Asmaa 2024. (A1) *Escherichia coli* (A2) *Klebsiella pneumoniae* (A3) *Proteus vulgaris* (A4) *Pseudomonas cepacia* (A5) *Pseudomonas fragi* (A6) *Enterobacter cloace* (A7) *Enterobacter aerogenes* (A8) *Serratia liquifaciens* (A9) *Staphylococcus aureus* (A10) *Staphylococcus epidermidis* (A11) *Streptococcus pyrogenes* (A12) *Enterococcus fecalis* (A13) *Bacillus subtilis*. (Mean values ± SD represented in bar graphs with asterisk indicate significant differences (*P* < 0.05; *n* = 3)

The use of static biofilm formation screening allowed this research to confirm that the tested strains could produce strong biofilms. The findings of MIC tests exhibited that the metabolites of *A. frequens* inhibited biofilm development in all of the strains that were examined. *Klebsiella pneumoniae* and *Bacillus subtilis* had the greatest percentages of biofilm inhibition at 66.7 and 64.8%, respectively, followed by *Pseudomonas fragi* (49.4%), *Staphylococcus aureus* (40.9%), and *Proteus vulgaris* (43.8%). The percentage of suppression of biofilm formation in the remaining strains varied from 16.4% for *Enterobacter cloacae* to 37.8% for *Escherichia coli* (Fig. 8).

### Zeta potential measurements

All studied Gram-positive and Gram-negative bacteria exhibited negative zeta potential values. Due to the presence of an additional negatively charged lipopolysaccharide layer (LPS). Gram-negative bacteria exhibit higher zeta potential values compared to Gram-positive bacteria. *S. aureus* exhibited a zeta potential of − 19.31 mV, whereas *S. pyrogenes* displayed a zeta potential of − 3.10 mV. In contrast, *E. coli* and *K. pneumoniae* presented zeta potential values of − 10.4 mV and − 16.1 mV, respectively. The zeta potential values for *E. coli* and *K. pneumoniae* were measured at − 8.79 mV and − 8.73 mV, respectively, following treatment with pigmented fungal extracts. The zeta potential values for *S. aureus* and *S. pyrogenes* were measured at − 16.7 mV and − 16.2 mV, respectively. Following treatment with the metabolites of *A. frequens*, the particle sizes of *E. coli*, *K. pneumoniae*, *S. aureus*, and *S. pyrogenes* diminished dramatically from 6996, 6366, 4483, and 5198 nm to 444.5, 411.3, 1038, and 921.1 nm, respectively. The PDI values for treated *E. coli*,

*K. pneumoniae*, *S. aureus*, and *S. pyrogenes* significantly diminished from 0.968, 1.0, 0.883, and 1.0 N/A, respectively, to 0.560, 0.548, 0.822, and 0.947 N/A, respectively (Table 5).

**Table 5:** Particle size, polydispersity index, and zeta potential of some selected pathogenic bacteria before and after treatment with the crude pigment produced by *A. frequens* strain Asmaa 2024

Bacterial species		Tested parameter	Mean ± S.D.	Unit*
*Escherichia coli*	Control	Particle size	6996 ± 832.1	nm
		PDI	0.968 ± 0.056	–
		Zeta potential	− 10.4 ± 0.252	mV
	Treated	Particle size	444.5^*^ ± 9.815	nm
		PDI	0.560^*^ ± 0.027	–
		Zeta potential	− 8.79^*^ ± 0.442	mV
*Klebsiella pneumoniae*	Control	Particle size	6366 ± 578.3	nm
		PDI	1.0 ± 0.0	–
		Zeta potential	− 16.1 ± 0.451	mV
	Treated	Particle size	411.3^*^ ± 8.529	nm
		PDI	0.548^*^ ± 0.021	–
		Zeta potential	− 8.73^*^ ± 0.0436	mV
*Staphylococcus aureus*	Control	Particle size	4483 ± 200.9	nm
		PDI	0.883 ± 0.117	–
		Zeta potential	− 9.31 ± 0.550	mV
	Treated	Particle size	1038^*^ ± 30.99	nm
		PDI	0.822 ± 0.087	–
		Zeta potential	− 16.7^*^ ± 1.44	mV
*Streptococcus pyrogenes*	Control	Particle size	5198 ± 514.7	nm
		PDI	1.0 ± 0.0	–
		Zeta potential	− 3.10 ± 0.546	mV
	Treated	Particle size	921.1^*^ ± 50.18	nm
		PDI	0.947 ± 0.083	–
		Zeta potential	− 16.2^*^ ± 0.929	mV

Unit*, nm: Nanometres; mV: Millivolts

### SEM analysis of *K. pneumoniae* bacterial cells

**Fig. 9:**
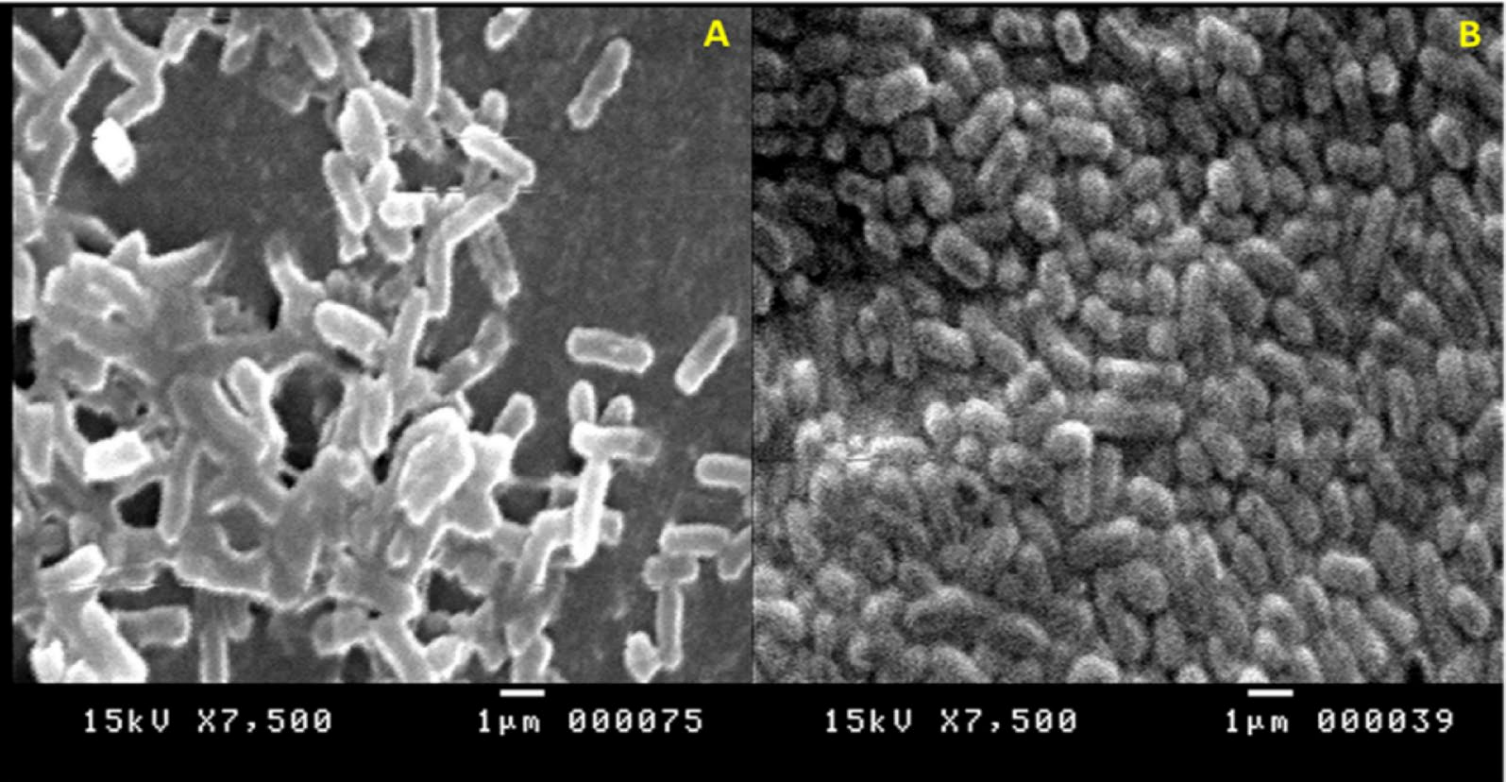
SEM images of *K. pneumoniae* (**A**) Untreated cells (**B**) Treated cells with the crude pigment produced by *A. frequens* strain Asmaa 2024 at Scale bar = 1 μm

Scanning electron microscopy was used to study how *A. frequens* metabolites affected the bacterial cell shape. In contrast to their normally noticeable wall structure, the present findings showed that *K. pneumoniae* cells underwent significant unfavorable morphological alterations. From their original rod shape, cells contracted and became more curved. As the cells engorged and released their protoplasm during disintegration, amorphous forms appeared (Fig. 9).

### Anticancer efficiency of the *A. frequens*’ crude pigment

**Fig. 10:**
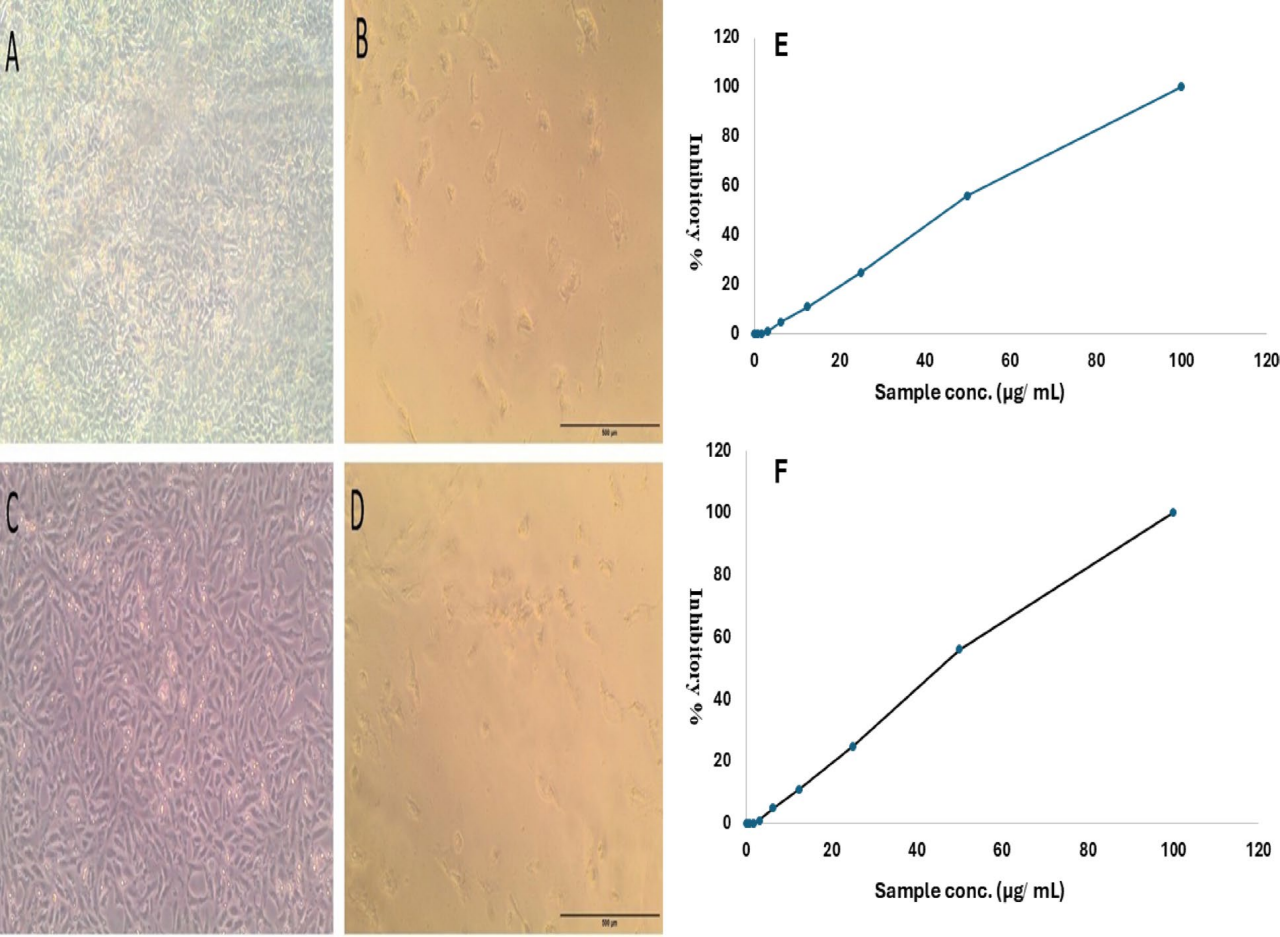
Cytotoxicity effect of *A. frequens* crude pigment on some tumor cell lines. Untreated control A549 cell line (**A**) and HOS cell line (**C**). Treated A549 (**B**) and HOS (**D**) at Scale bar = 500 μm. Inhibitory % of the pigment in different concentrations against the A549 cell line (**E**) and HOS cell line (**F**)

An MTT reduction experiment was performed with 100 to 0.78 µg/mL concentrations to assess the in vitro efficacy of *A. frequens* extracellular pigment against three distinct human cancer cell lines, and the inhibitory % increased with increasing pigment concentration (Fig. 10E, F). A concentration of 77.1 µg/mL of the *A. frequens* pigment resulted in 50% cell mortality (IC_50_), and 99.9 µg/mL induced 90% mortality (IC_90_) in lung adenocarcinoma (A549) cell lines. *A. frequens* pigment dramatically reduced the viability of the osteosarcoma cell line (HOS), where concentration, 43.3 µg/mL, is sufficient to inhibit 50% of the osteosarcoma cell lines (IC_50_), and 70.1 µg/mL suppresses 90% of the bone tumor cells (IC_90_). The skin cancer cell lines (A431) exhibited no alterations (Table 6). Untreated cells of A549 and HOS exhibited a dense, confluent monolayer with elongated and spindle-shaped morphology, indicating healthy growth and high viability (Fig. 10A, C), respectively. A549 and HOS malignant cells exposed to *A. frequens* crude pigment at 100 µg/mL showed marked morphological alterations, including reduced cell density, rounding, shrinkage, and detachment from the surface, consistent with cytotoxic and apoptotic effects (Fig. 10B, D), respectively.

**Table 6:** Anti-tumor efficiency of *A*. *frequens* pigmented secondary metabolites against 3 types of tumor cell lines

Tested tumor cell line	IC_50_ (µg/mL)	IC_90_ (µg/mL)	Remarks
Lung carcinoma cell line (A549)	77.1	99.9	71.3% at 100ppm
Osteosarcoma cell line (HOS)	43.3	70.1	65.2% at 100ppm
A431 (Skin cancer)	–	–	53.7% at 40 µl
Negative control	–	–	0%

IC_50_: lethal concentration of *A*. *frequens* pigmented secondary metabolites that kills 50% of the tested cell lines

IC_90_: lethal concentration of *A*. *frequens* pigmented secondary metabolites that kills 90% of the tested cell lines

- : means not effective

## Discussion

In recent years, fungi have become significant, environmentally sustainable suppliers of natural colors due to their effortless processing, rapid proliferation in economical substrates, and development that is unaffected by climatic conditions. In this study, among the tested submerged fermentation media, PDB proved to be the most suitable medium for fungal pigmentation, followed by CzB and YDPB supported pigment production by the lowest number of the tested fungi. The variation in pigment biosynthesis in the 3 selected media may be attributed to differences in nutrient availability within each medium, Narendrababu and Shishupala [48]. The obtained data were in complete agreement with a contemporary work by Mwaheb et al. [49], who reported that the highest pigmentation from *Fusarium verticillioides* AUMC 15,934 was detected on PDB medium. *Aspergillus terreus* strain LCM8 produced 300 mg/L of melanin pigment on PDB after 2 weeks and can bioremediate soil containing Cadmium (Cd) [50].

The current investigation revealed that *A. frequens* strain Asmaa 2024 was the most proficient producer of extracellular pigmented secondary metabolites. *Aspergillus* consistently produced the largest number of secondary metabolites with extracellular pigmentation. Extracellular pigments are preferred in several investigations because of their stability, affordability, and ease of downstream processing [51]. *Aspergillus*, *Penicillium*, *Fusarium*, and *Monascus* can synthesize a variety of pigments, including melanins, quinones, sunchokes, carotenoids, flavones, phenazines, anthraquinone, and azaphilones [1, 52–54].

The reddish-brown pigment extracted from *A*. *frequens* showed maximum pigment absorbance at a wavelength of 500 nm. This finding closely aligns with previous investigations [55, 56], which found that the red pigment azophilone extracted from *Talaromyces amestolkiae* showed maximum absorbance at 500 nm wavelength. Azaphilones are among the most promising classes of fungal pigments for industrial applications, distinguished by their vibrant yellow, orange, and red hues with maximum absorbance of red pigments at 500 nm [55, 57]. Although several previous studies have mentioned azaphilone production by many *Aspergillus* species, where six novel structures of azaphilone, Azanigerones A-f, have been detected in *Aspergillus niger*, indicating the azaphilone biosynthetic pathway in *Aspergillus* [58]. Penicitrinol Q (24), a dimeric azaphilone type, was extracted from *A*. *terreus* isolated from *Pinellia ternate* [59]. Three novel azaphilones, sassafrin E, sassafrin F, and sassafrinamine A, were generated by *Aspergillus neoglaber* [60]. This study is the first to investigate the production of colorful secondary metabolites by *A. frequens*.

The current findings indicated that PDB was the preferred medium for fungal growth and pigmentation, with no correlation between pigment intensity and biomass output in the tested isolates. Narendrababu and Shishupala [48] confirmed similar results that the high growth and pigment intensities of *Aspergillus* and *Penicillium* sp. were observed on PDB medium. The presence of functional groups in *A*. *frequens* pigmented secondary metabolites has been detected using FTIR. One effective method for the quantitative study of oils, fats, and palm carotene is FTIR, which uses the mid-infrared spectrum [61]. In this study, the detected functional groups were, O-H stretching group, C-H stretching group, alkenes (C = C), and C = O of ketones and quinones, C-N or CH_3_ bending, C-O-C, and C-N in addition to the aromatic ring substitution and deformation at the fingerprint region. Aligning with the obtained spectra, Keenkan et al. [62] confirmed similar functional groups in *Talaromyces purpureogenus* KKP azaphilone red pigment. FTIR analysis of the extracted red pigment from *Penicillium purpurogenum* Li-3 demonstrated the presence of C═C bonds, N–H groups, C═O functional group, long chains of aliphatic CH₂, and C–O linkages [63].FTIR analysis of *P. marneffei* red pigment confirmed that the pigment was β-unsaturated carbonyl compounds with NH/OH, aliphatic CH groups, ether groups, and aromatic CH [64]. Similar functional groups, O-H stretching, C-N stretching, C = C stretching, and C-O stretching, were previously detected by FTIR analysis of melanin pigment extracted from *Penicillium citrinum* strain NP4 [1].

Analysis of the resultant reddish-brown extract by GC-MS revealed the presence of many hues. GC‒MS is a rapid technique for the study of pigments [65]. The obtained chromatogram showed that a vast amount of bioactive compounds were present, revealing a complex mixture of 32 compounds, many of which exhibit coloring properties consistent with polyketide-derived pigments. Among the detected metabolites, several key compounds, including 2,6-dimethyl-γ-pyrone (pale yellow), are characteristic intermediates associated with the azaphilone biosynthetic pathway [70]. Azaphilones are well-known fungal polyketides characterized by a highly oxygenated pyranoquinone core, which undergoes diverse modifications, including amino and sulfur conjugations to yield pigments of variable colors: yellow, orange, and red, making them a promising pigment for cosmetics, dying, and printing applications [50, 58]. In the current investigation ((Z, Z)-1 H-Pyrrole-3-Propanoic Acid, 5-[(4-Ethenyl-1,5-Dihydro-3-Methyl-5-Oxo-2 h-Pyrrol-2-Ylidene)Methyl]-2-[[5-(Methoxycarbonyl)-3-(3-Methoxy-3-Oxopropyl)-4-Methyl-2 h-Pyrrol-2-Ylidene]Methyl]-4-Methyl-, Methyl Ester) was detected in the GC-MS analysis of the obtained *A. frequens* reddish brown pigment in consistent with a previous work by Bhardwaj et al. [64], who confirmed the presence of 1,1,3,3-tetramethyl-2,3-dihydropyrrole in the red pigment extracted from *Penicillium marneffei*. The detected fatty acids (hexadecanoic acid), generally identified as acyl donors in azaphilone biosynthesis, suggest the presence of polyketide metabolites in the extracted pigment of *A. frequens*. Furthermore, the two amino acids identified in the GC-MS analysis, l-alanyl-l-alanyl-l-alanine methyl ester and 2-methylpiperazine, which are reactive amine donors, suggest the formation of azaphilone derivatives through nitrogen conjugation, thereby increasing pigment stability and bioactivity. Orange azaphilones typically contain a pyran oxygen-bearing heterocycle that readily undergoes aminophilic reactions with peptides, nucleic acids, or amino acids, resulting in the substitution of oxygen by nitrogen and moving the pigment’s absorption from orange to red, and can modify its biological activities [66]. The different biological activities, including antimicrobial and antitumor of the detected compounds in *A*. *frequens* pigmented secondary metabolites extract are summarized in Table 3 [67–79].

The elemental and EDX analysis of the extracted pigment revealed high carbon and oxygen contents, supporting its organic nature and classification as an azaphilone-type secondary metabolite with moderate ratios of nitrogen and sulfur detected only by C, H, N, O, and S analysis. The notable nitrogen and sulfur ratios by the elemental analysis are attributed to that the elemental analysis provides a highly accurate quantitative estimation of the elements in the whole sample, while EDX offers less accurate detection of light elements like hydrogen and nitrogen, and reflects only the elemental surface composition of the sample [80, 81]. To our knowledge, this is the first report combining C, H, N, O, and S elemental analysis and EDX for fungal pigments generally and azaphilone in particular. The obtained EDX results are in consistent with Vega Gutierrez and Robinson [82], demonstrated the presence of only carbon and oxygen in red draconin, yellow, and xylindein fungal pigments with ratios ranging from 77 to 79% for C and 20–23% for O. SEM images indicated the polymorphic nature of the obtained fungal pigment particles with an average size of 40–184 nm. Vega Gutierrez and Robinson [82], reported that pigments obtained from *Chlorociboria aeruginosa* and *Scytalidium ganodermophthorum* formed an amorphous layer on wood, bamboo, and textiles. Melanin pigment extracted from *Hortaea werneckii* AS1 showed an average particle size (130–160 nm) [83].

This investigation revealed that the pigment of *A. frequens* effectively affected thirteen hazardous, multidrug-resistant biofilm-forming bacteria, with effective MBC between 11.5 and 16.7 mg/mL against Gram-positive bacteria. Interestingly, concentrations between 4.5 and 16.7 mg/mL were sufficient as MBC against Gram-negative bacteria. Azaphilones are widely recognized for their diverse biological activities, including antimicrobial, cytotoxic, anticancer, antiviral, and anti-inflammatory effects [84]. In line with this context, two types of azaphilones extracted from *(A) terreus*, Penctrimertone, effectively suppressed *(B) subtilis* with MIC of 4 µg/mL. Another type of azaphilones, penicitrinol Q, exhibited a promising inhibitory action against *S. aureus*, *B. subtilis*, and *P. aeruginosa* with MIC values of 4.3, 6.2, and 11.2 µg/mL, respectively [59], and in contrast to our obtained data, the MIC values of Gram-positive bacteria were lower than those of Gram-negative bacteria. Azaphilones, rubiginosin- and rutilin showed weak to moderate antibacterial efficacy against *Bacillus subtilis* and *Staphylococcus aureus* [85]. In comparison with a recent work by Narendrababu and Shishupala [86], the antibacterial effect of *A. frequens* crude pigments was higher compared with nine types of *A. nidulans* crude pigments, where *(A) nidulans* pigments affected only Gram-positive bacteria, such as *(B) subtilis* and *S. aureus*, whereas no inhibition was noticed against the Gram-negative bacteria. Based on the chemical profiling of the fungal pigment, several bioactive compounds were identified that may explain the observed antibacterial efficacy by disrupting bacterial cell membranes and inhibiting essential enzymatic processes, resulting in growth inhibition. Fungal pigments have an antimicrobial impact because of their ability to induce oxidative stress and reactive oxygen species (ROS) within bacterial cells, inhibiting growth [87]. Furthermore, the highest effect on *E. coli* and *P. vulgaris* may be due to the weakness of these strains with limited antibacterial resistance, plus certain pigments might interact with or alter the components of the bacterial cell wall and membrane, increasing their permeability and making the bacteria more susceptible to the pigment’s effects [88]. The antibacterial efficacy of some *A. frequens* GC-MS detected compounds has been previously described, such as ((Z, Z)-1 H-Pyrrole-3-Propanoic Acid, 5-[(4-Ethenyl-1,5-Dihydro-3-Methyl-5-Oxo-2 h-Pyrrol-2-Ylidene)Methyl]-2-[[5 (Methoxycarbonyl)-3-(3-Methoxy-3-Oxopropyl)-4-Methyl-2 h-Pyrrol-2-Ylidene]Methyl]-4-Methyl-, Methyl Ester) [69], 3-(2-Aminophenyl)Isocoumarin [71], 3-(4-Chlorophenyl)-4,6-Dimethoxy-1-(prop-2’-e nyl)Indole-7-Carbaldehyde [73], 4-Amino-1-pentanol [76, 77], and 6-Aza-5,7,12,14 tetrathiapentacene [78].

The pigment of *A. frequens* in this investigation displayed substantial efficiency against bacteria that had previously formed biofilms. Biofilms pose considerable therapeutic challenges, as pharmaceuticals struggle to penetrate the extracellular polymeric material [89]. These results demonstrated that *Klebsiella pneumoniae* and *Bacillus subtilis* exhibited the highest biofilm inhibition rates of 66.8% and 64.8%, respectively. A new research field is developing that concentrates on antibacterial medicines aimed at bacterial surfaces, driven by mounting evidence of drug-resistant biofilms from pathogens and a shortage of antibiotics [90]. Essential agents demonstrating substantial bactericidal effectiveness and a consequent decrease in the likelihood of resistance emergence are those that act on the bacterial cell surface [91]. Phospholipids, teichoic acid, and both basic and acidic functional groups linked to lipopolysaccharides (LPSs) are present on the surfaces of all bacteria, regardless of their cell wall composition [92]. Studies by Borrok et al. [93]. and Hong and Brown [94] demonstrated that these groups influence the electrostatic characteristics of cells, hence modulating bacterial adhesion. Halder et al. [90]. asserted that the majority of bacteria display a negative surface charge, as defined by their zeta potential. Saito et al. [95]. recorded the potential disparity between the bacterial cell surface and the aqueous solution. Tokumasu et al. [96] noted that this potential is crucial for maintaining cellular activity and transmitting information about surface characteristics.

Fungal pigments interacted electrostatically with bacterial cell surfaces, influencing the zeta potential, altering surface permeability, and ultimately resulting in cell death. In this study, this interaction significantly reduced the zeta potential values for Gram-negative bacteria, specifically *E. coli* and *K. pneumoniae*, from − 10.4 to − 16.1 mV to − 8.79 and − 8.73 mV, respectively. The zeta potential of Gram-positive bacteria in this work, such as *Staphylococcus aureus* and *Staphylococcus pyogenes*, markedly increased from − 9.31 to − 3.10 mV to − 16.2 mV, respectively, upon contact. Some chemicals must first neutralize the bacterial membrane to exhibit antibiotic activity [97]. Increasing zeta potential post-treatment of Gram-negative bacteria generally refers to the degree of repulsion between bacterial cells in the liquid media. Furthermore, Gram-negative bacteria have a more negative zeta potential than Gram-positive bacteria because their outer membrane contains negatively charged lipopolysaccharides (LPS). Decrease after treatment due to attraction and potential for aggregation due to the reaction of bacteria with pigment, and disruption occurs in the bacterial cell wall and cell membrane. While in Gram-positive bacteria, pigment may be deposited on the bacterial surface. This deposition can introduce a more positive charge onto the negatively charged bacterial surface [90].

This study reported the diameters of untreated *E. coli* (6996 nm), *K. pneumoniae* (6366 nm), *S. aureus* (4483 nm), and *S. pyogenes* (5198 nm). On the other hand, the treated cells reduced in size to 444.5, 411.3, 1038, and 921.1 nm, respectively. The substantial decrease may be ascribed to bacterial cell death and lysis, which reduce autoaggregation. Bacteria can participate in autoaggregation or autoagglutination, as well as adhere to host cells. This trait is exhibited by various bacteria. The macroscopic observation of bacterial aggregates settling at the bottom of culture tubes indicated autoaggregation [98].

In this research, due to bacterial cell mortality, diminished aggregation, and reduced heterogeneity, the PDI values of treated bacteria, including *E. coli*, *K. pneumoniae*, *S. aureus*, and *S. pyogenes* decreased from 0.968, 1.0, 0.883, and 1.0 N/A to 0.560, 0.548, 0.822, and 0.947 N/A, respectively. The polydispersity index (PDI) is a method to assess size-dependent heterogeneity in a sample. Consequently, sample aggregation or the variation in sample sizes may transpire [99]. The bulk material is compacted or agglomerated, exhibiting significant variance in particle sizes. Values beyond 0.7 signify an exceptionally broad spectrum of sizes.

In this study, treatment of 3 types of human malignant cells with *A. frequens* pigment resulted in in vitro anticancer activity against A549 and HOS cell lines, with IC₅₀ values of about 77.1 µg/mL and 43.3 µg/mL, respectively. On the other hand, the A431 skin cell line demonstrated resistance to the evaluated pigment. Fungal pigments showed hopeful anti-tumor potential that suppressed the cancerous cells by many mechanisms, like triggering apoptosis by activating caspase-3 and lowering the potential of the mitochondrial membrane, suppressing angiogenesis, generation of ROS, oxidative stress, inflammation, and activation of signaling pathways (NF-κB pathway) [99, 100]. Consistent with the recorded findings, eight chlorinated azaphilone metabolites were obtained from the marine fungus *Chaetomium* sp. NA-S01-R1 and assessed for their anti-tumor potential; seven exhibited activities against A549 cells, with IC₅₀ values ranging from 15.2 ± 0.9 to 40.0 ± 0.3 µM [102]. Penidioxolone C, azaphilone type isolated from *P. sclerotiorum*, demonstrates moderate cytotoxicity against A549 lung carcinoma cells (IC₅₀ = 60.16 ± 0.26 µM), along with activities against multiple other cancer lines [103]. In a recent investigation, melanin pigment extracted from fungi isolated from different macroalgae significantly suppressed lung adenocarcinoma (A549), with the lowest IC50 being detected in *Alternaria alternata*, while facilitating the metabolism and proliferation in normal A549 cell lines confirming the selective toxicity of fungal pigments and observed the same detected malformations in the present study such as shrinking, rounding, and detachment in treated malignant cells [104]. The anticancer potential of some *A. frequens* GC-MS detected compounds has been reported in previous studies, such as, ((Z, Z)-1 H-Pyrrole-3-Propanoic Acid, 5-[(4-Ethenyl-1,5-Dihydro-3-Methyl-5-Oxo-2 h-Pyrrol-2-Ylidene)Methyl]-2-[[5 (Methoxycarbonyl)-3-(3-Methoxy-3-Oxopropyl)-4-Methyl-2 h-Pyrrol-2-Ylidene]Methyl]-4-Methyl-, Methyl Ester) [69], 3-(2-Aminophenyl)Isocoumarin [71], l-Alanyl-l-alanyl-l-alanine methyl ester [72], 3-(4-Chlorophenyl)-4,6-Dimethoxy-1-(prop-2’-e nyl)Indole-7-Carbaldehyde [73], and 4-Amino-1-pentanol [77]. In comparison with the cytotoxic potency of other *Aspergillus* pigments, rhodopin pigment extracted from *A. nidulans* was tested against HEp-2 tumor cells alone and after treatment with + 10 Gy γ-radiation and showed IC_50_ of about 208 µg/mL and 115 µg/mL, respectively [105]. This investigation has some limitations, as it tested cytotoxicity in vitro rather than in vivo, did not explore pigment-selective toxicity, and did not investigate the possible mechanism of action. These limitations should be thoroughly considered in forthcoming investigations.

## Conclusion

In this study, *A. frequens* for the first time produces a valuable azaphilone pigment with significant biological activities. The pigments inhibited 13 pathogenic bacteria at minimal bactericidal levels. Biofilm suppression was strong in *Klebsiella pneumoniae* (66.8%) and *Bacillus subtilis* (64.8%). Zeta potential, particle size, and PDI analyses corroborated antibacterial activity. The metabolites also significantly reduced HOS and A549 cell lines. So, these pigments can be utilized in the medical treatment of bacterial infections and for the suppression of tumor cell proliferation. Future studies will focus on detailed compound characterization, optimization, in vivo determination of the cytotoxic potential, evaluation of the selective toxicity, and determination of the antitumor mechanism.

## Data Availability

“The dataset generated and/or analyzed during the current study is available in the GenBank: *Aspergillus frequens* strain Asmaa 2024 internal transcribed spacer 1, - Nucleotide - NCBI; accession number PQ249413”.
